# A Research Agenda for Helminth Diseases of Humans: Diagnostics for Control and Elimination Programmes

**DOI:** 10.1371/journal.pntd.0001601

**Published:** 2012-04-24

**Authors:** James S. McCarthy, Sara Lustigman, Guo-Jing Yang, Rashida M. Barakat, Héctor H. García, Banchob Sripa, Arve Lee Willingham, Roger K. Prichard, María-Gloria Basáñez

**Affiliations:** 1 Queensland Institute of Medical Research, University of Queensland, Herston, Australia; 2 Lindsley F. Kimball Research Institute, New York Blood Center, New York, New York, United States of America; 3 Department of Schistosomiasis Control, Jiangsu Institute of Parasitic Diseases, Jiangsu, People's Republic of China; 4 High Institute of Public Health, Alexandria University, Alexandria, Egypt; 5 Department of Microbiology, Universidad Peruana Cayetano Heredia, Lima, Peru; 6 Tropical Disease Research Laboratory, Division of Experimental Pathology, Department of Pathology, Faculty of Medicine, Khon Kaen University, Khon Kaen, Thailand; 7 Special Programme for Research and Training in Tropical Diseases (TDR), World Health Organization, Geneva, Switzerland; 8 Institute of Parasitology, McGill University, Montreal, Canada; 9 Department of Infectious Disease Epidemiology, School of Public Health, Faculty of Medicine, Imperial College London, London, United Kingdom; London School of Hygiene & Tropical Medicine, United Kingdom

## Abstract

Diagnostic tools appropriate for undertaking interventions to control helminth infections are key to their success. Many diagnostic tests for helminth infection have unsatisfactory performance characteristics and are not well suited for use in the parasite control programmes that are being increasingly implemented. Although the application of modern laboratory research techniques to improve diagnostics for helminth infection has resulted in some technical advances, uptake has not been uniform. Frequently, pilot or proof of concept studies of promising diagnostic technologies have not been followed by much needed product development, and in many settings diagnosis continues to rely on insensitive and unsatisfactory parasitological or serodiagnostic techniques. In contrast, PCR-based xenomonitoring of arthropod vectors, and use of parasite recombinant proteins as reagents for serodiagnostic tests, have resulted in critical advances in the control of specific helminth parasites. The Disease Reference Group on Helminths Infections (DRG4), established in 2009 by the Special Programme for Research and Training in Tropical Diseases (TDR) was given the mandate to review helminthiases research and identify research priorities and gaps. In this review, the diagnostic technologies relevant to control of helminth infections, either available or in development, are reviewed. Critical gaps are identified and opportunities to improve needed technologies are discussed.

## Introduction

The technical limitations of currently available diagnostic methods for helminth infections impose significant constraints on current initiatives to control these infections, as discussed in other reviews of this collection [1], [2]. Appropriate diagnostic methodologies are required for: a) disease mapping to guide initiation and prioritisation of interventions; b) monitoring and evaluation (M&E) of ongoing interventions, and particularly for the prompt detection of possible emerging anthelmintic resistance; c) assessment of elimination of infection by elimination programmes as these approach termination; and d) case-based diagnosis for surveillance.

For each of these activities, the technical requirements for diagnostic tests differ and pose different technical challenges. Furthermore, for each helminth species, the biology of the parasite (life cycle, accessibility of parasite stages for parasitological diagnosis, body fluid appropriate for sampling, role of and need to sample vector or intermediate host), and of the parasite–host system (including age profiles of infection prevalence and intensity) impose different constraints on diagnostic capacity. In addition, intensity of infection is a critical determinant of transmission dynamics, morbidity, and disease burden. Thus, the need to assess infection burden is critical to understanding density-dependent regulatory mechanisms of parasite transmission and morbidity. Yet, with few exceptions, this critical parameter is difficult to quantify accurately with current diagnostic tools.

Not only are techniques for the diagnosis of individual infections important, but also techniques for assessment in communities and larger regions are necessary. In this respect, a major obstacle to the implementation of cost-effective control is the lack of accurate descriptions of the geographical distribution of infection. The effectiveness of large-scale integrated programmes for the control of neglected tropical diseases (NTDs) in general, and helminth diseases in particular, depends on appreciation of the geographical overlap between the different NTDs. However, in spite of being co-endemic in most countries, different NTDs can exhibit limited geographical overlap at sub-national scales, necessitating a more geographically targeted approach for their control. Therefore, techniques appropriate for the diagnosis and surveillance of helminth infections are essential for solving many of the control challenges described in other articles of this collection [1], [2].

The range of diagnostic tools that are available at present can be classified into: 1) parasitological tests, where the parasite (or more frequently parasite transmission stages), sampled from appropriate tissues or body fluids, is directly visualised using a microscope (not necessary for visualisation of large nematodes such as adult *Ascaris*); 2) serological assays, where parasite-specific antibodies are detected in serum samples; 3) antigen detection tests, where a parasite biomarker is detected; 4) molecular diagnosis, where parasite nucleic acid is detected; and 5) other specific tools for parasite detection in arthropod vectors or snail (or other) intermediate hosts.

As noted above, there are important differences in the specific requirements for diagnostic tests for each helminth parasite, differences that are determined by the biology of the parasite and the control interventions that are currently deployed. Likewise, the critical gaps in diagnostic technology differ for each parasite species. In this review, a product of the discussions held by the Disease Reference Group on Helminth Infections (DRG4) established in 2009 by the Special Programme for Research and Training in Tropical Diseases (TDR), available diagnostic tests for the six helminth human infections, within the remit of the DRG4, are considered in the context of the specific technical requirements of diagnostic tests needed for control of these parasites, and critical gaps in diagnostic technology are identified. The technical issues related to the performance of tests using parasitological, serological, antigen detection, molecular detection, and other specific tools for diagnosis in vectors and intermediate hosts are detailed in the following supplementary files: Text S1 for soil-transmitted helminthiases; Text S2 for filarial infections; Text S3 for schistosomiasis; Text S4 for hydatid disease; Text S5 for taeniasis/cystercosis; and Text S6 for food-borne trematode infections. A summary of the current diagnostic tools for monitoring and surveillance in control programmes is presented in Table 1. The use of these tests in the context of control and elimination activities is outlined in the sections below. Box 1 lists the abbreviations used in this paper. The desired attributes of diagnostic techniques required for the successful implementation of mass drug administration (MDA) programmes are listed in Box 2.

**Box 1. List of Abbreviations**

**APOC,** African Programme for Onchocerciasis Control

**CAA,** circulating anodic antigen

**CCA,** circulating cathodic antigen

**CFA,** circulating filarial antigen

**CHD,** cystic hydatid disease

**DDIA,** dipstick dye immunoassay

**DEC,** diethylcarbamazine

**DFID,** Department for International Development, United Kingdom

**DRG4,** Disease Reference Group in Helminth Infections

**FDA,** Food and Drug Administration, United States

**FECRT,** faecal egg count reduction test

**FIND,** Foundation for Innovative New Diagnostics

**GIS,** geographical information system

**ICT,** immunochromatographic test

**ITS,** internal transcribed spacer

**LAMP,** loop-mediated isothermal amplification

**LF,** lymphatic filariasis

**LIPS,** luciferase immunoprecipitation system

**MDA,** mass drug administration

**MDG,** Millenium Development Goal

**MDSS,** Medical Device Safety Service, European Union

**M&E,** monitoring and evaluation

**mf,** microfilariae

**NCC,** neurocysticercosis

**NTD,** neglected tropical disease

**PCR,** polymerase chain reaction

**PDIP,** Product Development and Implementation Partnership

**POC,** point-of-care

**RAPLOA,** rapid assessment procedure of loiasis

**R&D,** research and development

**REA,** rapid epidemiological assessment

**REMO,** rapid epidemiological mapping of onchocerciasis

**SAE,** severe adverse event

**SEA,** soluble egg antigen

**STH,** soil-transmitted helminthiasis

**TB,** tuberculosis

**TDR,** Special Programme for Research and Training in Tropical Diseases

**WHO,** World Health Organization

**Box 2. Five Desirable Attributes of Diagnostic Tests Required for Helminth Control Programmes**

- Scalable cost-effective tests for individual diagnosis. Ideally these should be suitable for point-of-care (POC) use, or in environments where technical resources are limited
- Tests suitable for large-scale surveys, for example for epidemiological surveillance
- Tests capable of quantifying infection intensity (worm burden) reliably, a diagnostic parameter that is critical when dealing with helminth infections
- Tests enabling quantification of suboptimal responses to control interventions including prompt detection of drug resistance
- High specificity and sensitivity as infection prevalence and intensity decrease throughout intervention, enabling to assess programme end points

**Table 1:** Diagnostic Tools Available for Monitoring and Surveillance in Control Programmes for Human Helminthiases.

Helminth Infection	Stage of the Control Programme			References
	Early	Advanced	Final and End Points	
Onchocerciasis	Nodule palpation for detection of onchocercomata, skin-snipping for detection, and counting of mf in skin snips	Skin snipping sensitivity decreases; DEC patch test; PCR-based monitoring of simulid populations; Ov-16 card test	Xenomonitoring via fly feeding/recording microfilarial uptake. Serology in untreated children; PCR and DEC patch test; Ov-16 card test	[106], [107]
Lymphatic filariasis	Blood smears for detection and counting of mf in blood; CFA for bancroftian filariasis. Rapid dipstick for antibody (Ab) detection in brugian filariasis	PCR-based assays for *W. bancrofti* and *B. malayi* in blood; PCR-based monitoring of mosquito populations	Monitoring infections in mosquitoes and anti-filarial Ab levels in children as indicators of local transmission for making decisions about programme end points	[108]–[110]
Soil-transmitted helminthiases	Quantitative egg counts using validated methodology such as Kato-Katz (KK) test	Infections become lighter and more difficult to detect. Egg concentration techniques, e.g., FLOTAC likely to be required to detect light infections	Increasing proportion of unfertilised *Ascaris* eggs could indicate declining mating probability (unfertilised eggs often missed by KK). Need for highly sensitive diagnostic methods	[11]–[13]
Intestinal schistosomiasis due to *S. mansoni*	As above	Need to validate PCR-based diagnostic assays in low-transmission areas	Elimination of infection reservoir rarely attempted	[111]
Intestinal schistosomiasis due to *S. japonicum*	Initial screening for antibodies in indirect haemagglutination assay (IHA), subsequent testing with KK of the seropositive results	Seroprevalence determined by IHA can be much higher than prevalence in stool-based PCR; hatching and KK tests	New algorithms for treatment in low intensity areas; PCR may replace KK in such algorithms. Surveillance in snails; sentinel mice	[111]–[114]
Urinary schistosomiasis (*S. haematobium*)	Urine filtration for detection and counting of eggs	Need to improve urine circulating antigen test for use in low-transmission areas	Find and treat cases in both active surveillance and health care settings	[111]

## Soil-Transmitted Helminthiases

Current control programmes for the intestinal nematodes or soil-transmitted helminthiases (STHs), namely, those caused by *Ascaris lumbricoides*, hookworm (*Necator americanus* and *Ancylostoma duodenale*), and *Trichuris trichiura* (and to a lesser extent by *Strongyloides stercoralis*) are focused on morbidity control through community-based deworming programmes, particularly among school-age children by annual or twice annual distribution of a single dose of a broad spectrum benzimidazole anthelmintic, either albendazole or mebendazole [2], [3]. Although parasitological diagnosis can be readily undertaken (see below), a number of critical deficiencies in diagnostic capacity exist. These include the absence of well-validated and practical methodologies to measure infection intensity (egg count is taken as a proxy for this but this approach has limitations), and validated cutoffs for drug efficacy monitoring [4]. A detailed discussion of the strengths and weaknesses of diagnostic tests appropriate for diagnosis of STHs is presented in Text S1, and a summary of the use of current STH diagnostic tests for specific objectives is presented in Table 2. The research priorities for STH diagnosis are summarised in Box 3.

**Box 3. Research Priorities for Helminth Diagnostics**

**Soil-Transmitted Helminthiases**

1. Improve and validate tools for quantifying intensity of infection (worm burden), including both biomarkers (coproantigen) and molecular methods (PCR-based)
2. Refine/develop methods to assess response to treatment for monitoring of drug efficacy
3. Refine methods for more accurate quantification of egg counts
4. Develop and validate methods for quantifying parasite genetic diversity and population structure

**Filarial Infections**

1. Devise diagnostic tools (e.g., biomarkers of active infection) to assist elimination. Most available diagnostic methods are too insensitive for M&E and surveillance once infection prevalence falls to very low levels
2. Develop circulating antigen tests for detection of *O. volvulus*, *L. loa*, and *B. malayi* infection that can be used in combination with current tools to assess active infection
3. Identify and validate informative molecular markers for detection of heritable changes in drug efficacy, and develop cost-effective tools to detect such changes
4. Develop and validate methods for quantifying parasite genetic diversity and population structure

**Schistosomiasis**

1. Refine antigen tests and optimise algorithms for their combination with existing diagnostic assays including parasitological, immunological, and ultrasound tools
2. Develop assays for assessment of worm burden in areas of varying endemicity
3. Identify and standardise markers and assays for resistance to praziquantel for monitoring of drug efficacy
4. Improve and standardise protocols for monitoring of infection in humans and snails by molecular methods
5. Develop and validate methods for quantifying parasite genetic diversity and population structure

**Cystic Hydatid Disease**

1. Develop rapid, sensitive, and specific point-of-care (POC) tests for canine echinococcosis

**Taeniasis/Cysticercosis**

1. Develop rapid, sensitive, and specific POC tests for human taeniasis
2. Develop sensitive and specific tests for detection of viable *Taenia solium* infections in pigs
3. Devise quantitative tests to determine intensity of infection

**Food-Borne Trematodiases**

1. Develop species-specific monoclonal antibodies suitable for use in specific coproantigen detection tests
2. Validate effective and inexpensive molecular methods for differentiation of fluke species with similar egg morphology

**Table 2:** Diagnostics Available for Soil-Transmitted Helminthiases.

Diagnostic Procedure and Objective	Species of Intestinal Nematode	
	*Ascaris lumbricoides* (roundworm), *Trichuris trichiura* (whipworm), *Necator americanus/Ancylostoma duodenale* (hookworm)	*Strongyloides stercoralis* (threadworm)
Stool microscopy with or without concentration step^a^	✓	+/−
Coproculture	Harada Mori for specific identification of hookworms	✓
Antibody detection	N/A	✓
PCR and antigen detection	----Experimental----	
Assessment of infection intensity	Quantitative fecal egg count; PCR (experimental)	N/A
Assessment of drug efficacy	Reduction in stool egg counts (FECRT)	Negative coproculture; decline in antibody titer
Mapping	Stool microscopy	Antibody detection
Elimination	N/A	N/A

a By sedimentation (e.g., formalin-ethyl acetate sedimentation) or flotation (e.g., ZnSO4).

✓, Available or method of choice; N/A, not available; +/−, acceptable but not ideal.

### Infection Intensity

For the STHs, intensity of infection is a major determinant of morbidity, infection dynamics, and response to therapy, as well as a crucial variable for epidemiological modelling [5]. Further, aggregation of infection, whereby a minority of individuals harbour very heavy infection loads but the majority of the host population harbours light or moderate infection, is a major factor to consider in epidemiological studies and chemotherapy efficacy trials, as well as in efforts to reduce prevalence. Intensity of infection would be best assessed by enumerating adult worm numbers. While this can be achieved by use of a cathartic in conjunction with an anthelmintic, preferably one that paralyses adult worms, such as pyrantel pamoate or levamisole, followed by enumeration of parasites in collected faeces, this is not an easy test to perform, and is unreliable for hookworms and whipworms because of poor to moderate, and variable, efficacy of pyrantel and levamisole against these parasites [6]. Therefore, most studies and surveys have relied on quantitative egg counting.

Of the quantitative methods described for egg counting, the Kato-Katz method [7], originally developed for detection and quantification of *Schistosoma mansoni* eggs in human faecal samples, is the most widely used method, and has undergone a number of validation studies [8], [9]. This test has some methodological problems due to the fact that different helminth eggs have different clearing times and viability. Hookworm eggs in particular are subject to lysis if the slides are not examined within 30 minutes [10]. A preferred quantitative flotation technique used in veterinary practice is the McMaster test [4]. It is less well studied in the human STH infections. In a recently undertaken study where these two methods were compared, the accuracy of the two tests was approximately equivalent [4]. The McMaster method has the drawback of not being suitable for community studies where intestinal schistosomiasis is co-endemic (China, Africa, South America). Recently, a purpose-built flotation apparatus (FLOTAC) has been designed and tested for the purpose of improving the quantitative analysis of faecal egg counts [11]. Although the FLOTAC method is more sensitive than the Kato-Katz technique [12], [13], particularly for hookworm eggs, it requires a centrifuge and is relatively low-throughput.

### Application of Diagnostics for Assessment of Response to Anthelmintic Chemotherapy including Detection of Anthelmintic Resistance

Although quantitative measurement of infection intensity by egg counting before and after treatment is the best available method for assessment of anthelmintic efficacy, this approach suffers from a number of significant drawbacks. For example, the relationship between worm burden and egg output is not linearly proportional, with density-dependent fecundity resulting in the potential underestimation of adult worm burden, for example in *A. lumbricoides* and hookworm infection [14]–[16].

The weaknesses in study design and consequent uncertainties in measures of STH anthelmintic drug efficacy have been reviewed [2], [17], [18]. In a recent review, specific guidelines regarding study design, including intervals between testing and expected clearance thresholds, have been recommended [2]. A specific problem is the lack of inter-study standardisation of diagnostic tests used. Two other specific issues pertain to technical aspects of diagnosis: detection of low level infection, especially when a single stool sample is examined using methods such as Kato-Katz, and a lack of standardisation of the assessment of reduction in infection intensity as determined by reduction in faecal egg counts. While direct parasitological tests are relatively sensitive, their performance is suboptimal at low intensity of infection, and therefore a single negative stool examination for helminth eggs is liable to result in Type II errors in evaluation of trial outcomes, i.e., misclassification of decreased drug efficacy as intact due to failure to detect persistent, low level infection following anthelmintic therapy. As discussed above, while there is no well-accepted measure of intensity of infection, trials have generally reported some measure of reduction in egg count, but not in a standardised fashion. In veterinary practice, significant work has been undertaken to standardise assessment of anthelmintic drug efficacy [19], specifically in standardising all aspects of the faecal egg count reduction test (FECRT). As noted above, assessment of drug efficacy by reporting reduction in egg counts is, in addition, subject to confounding due to the effect of density-dependent fecundity on egg counts [16]. There may also be considerable geographic heterogeneity in the strength of density-dependent constraints on worm fecundity [14].

Pilot phenotypic tests of anthelmintic response have been developed for hookworms, taking advantage of the characteristic of hookworm eggs, whereby eggs passed in the faecal stream mature into infective larvae over a number of days [20]–[22]. While there has been some work undertaken to standardise such tests [23], significant work remains to be done. For *T. trichiura* and *A. lumbricoides*, it has not been possible to develop such phenotypic tests due to the lack of egg hatch in the external environment, and an inability to readily assess parasite viability ex vivo.

Some work has been conducted to develop molecular methods to genotype intestinal nematode parasites for genetic markers of anthelmintic resistance [24]. Efforts have largely concentrated on genotyping for specific polymorphisms in the β-tubulin gene that are recognised to be associated with anthelmintic resistance in a range of veterinary nematodes, specifically mutations in the β-tubulin gene at codons 200, 167, and 198. Assay methodologies have been developed for *A. lumbricoides* [25], hookworms [26], [27] and *T. trichiura* 25,28, with resistance-associated mutations being found in some populations of these soil-transmitted nematodes. This suggests that selection for resistance to anthelmintics such as albendazole and mebendazole can occur, and may help explain the sometimes low and variable efficacy of these anthelmintics against human STH infections (see [2], [24] for recommended cure efficacies). However, the relationship between the presence and frequency of these specific mutations and drug response phenotypes has not yet been fully characterised, and should become an important research priority so that fast and sensitive genotyping analyses, used on pooled egg samples, can provide surveillance tools for anthelmintic resistance.

### Application of Diagnostics for Disease Mapping, Surveillance, and Mathematical Modelling

Infection mapping of STHs has progressed well, and readers are referred to the http://www.thiswormyworld.org/ website. Although mathematical models of transmission (reviewed within this collection in [5]) have been developed, they may not account for current difficulties in accurately quantifying worm burden, a consideration that is seldom recognised.

## Filariases

The programmes to eliminate onchocerciasis from the Americas and lymphatic filariasis (LF) on a global basis have highlighted specific deficiencies in the diagnostics for the filariases, particularly in elimination settings. Some relate to the problem of surveillance when infection prevalence falls to low levels. In such settings, most available methods are too insensitive, even if active case detection were logistically feasible. Other specific issues include prediction of the risk of severe adverse events (SAEs) when individuals with high grade microfilaraemia with *Loa loa* are administered antifilarial chemotherapy, and detection and management of changes in drug efficacy that may indicate emerging resistance. A detailed discussion of the strengths and weaknesses of diagnostic tests appropriate for diagnosis of filariasis is available in Text S2, and a summary of current diagnostic tests for filariases is presented in Table 3. The research priorities for filarial diagnosis are summarised in Box 3.

**Table 3:** Diagnostics Available for Filarial Infections.

Objective	Lymphatic Filariasis		Onchocerciasis	Loiasis
	*Wuchereria bancrofti*	*Brugia malayi*	*Onchocerca volvulus*	*Loa loa*
Parasitological diagnosis	Blood filtration for microfilariae (mf)Ultrasound		Skin snipNodule palpationDEC patch test	Blood filtration for microfilariae
Antibody detection	✓	✓	✓	✓
Antigen detection	✓	–	–	–
PCR	Molecular xenomonitoring (PCR on mosquito/fly vectors)			–
Assessment of infection intensity	mf load in bloodAntigen level	mf load in blood	mf load in skinNodule palpation	mf load in blood
Assessment of drug efficacy	Disappearance of mf from blood at given times post-treatment Ultrasound changes in filarial worm nests Clearance of circulating antigen	Disappearance of mf from blood at given times post-treatmentUltrasound changes in filarial worm nests	Disappearance of mf from skin at given times post-treatment Ultrasound changes in nodules By nodule histology or embryogram examination	Disappearance of mf from blood
Mapping	Antigenaemia prevalence		REMO	RAPLOA
Elimination	Seroepidemiology Seroepidemiology Molecular xenodiagnosis of vectors (mosquito PCR)		SeroepidemiologyMolecular xenodiagnosis of vectors (blackfly PCR)	N/A

✓, Available or method of choice; N/A, not available; –, not available.

REMO, rapid epidemiological mapping of onchocerciasis; RAPLOA, rapid assessment procedure for loiasis.

### Infection Intensity

Infection intensity may refer to adult worm burden or microfilarial load. The relationship between microfilarial load, as assessed by quantification of blood microfilaraemia in LF and loaisis, or of microfilaridermia by skin snip in onchocerciasis, and the total adult parasite burden is at best semi-quantitative. In addition to limitations imposed by sampling methodology and measurement error, microfilarial load is significantly affected by immune-mediated parasite clearance mechanisms, and processes regulating the fecundity of adult worms. Assessment of adult worm burden in onchocerciasis by nodule palpation suffers from both sensitivity and specificity problems in addition to complications brought about by the aggregation of adult worms in nodules. Nodule prevalence in a sample of adult hosts has been used to estimate a measure of community infection for onchocerciasis, by relating nodule to microfilarial prevalence. While palpation of onchocercal nodules is the major tool for determination of infection prevalence and rapid epidemiological mapping of onchocerciasis (REMO) in areas of the African Programme for Onchocerciasis Control (APOC), where it is used for identification of communities at risk [29], and for selection of areas for mass ivermectin administration, it is only reliable in highly endemic areas. Its diagnostic precision is poor because of large intra- and inter-individual variability, and its ability to reflect changes in endemicity as a result of large-scale and prolonged control programmes is limited [30].

Circulating filarial antigen (CFA) in bancroftian filariasis is a product of adult worms, with the majority being produced by adult female worms. As it is very difficult to enumerate adult worm numbers in humans, the relationship between CFA level and parasite burden is difficult to establish, but has been achieved in relevant animal models [31]. In one study, where worm nests were enumerated by ultrasound, there was a non-significant association between CFA level and ultrasound signal [32].

### Application of Diagnostics for Assessment of Response to Anthelmintic Chemotherapy including Detection of Anthelmintic Resistance

Aside from assessment of changes in microfilarial counts following treatment, diagnostics for establishing cure of filariasis are generally lacking, and as discussed in [24], the available drugs for MDA are not generally macrofilaricidal (with the exception of prolonged courses of doxycycline to deplete *Wolbachia* endobacteria [33]). This is particularly problematic when drugs, with selective activity on the only life cycle stage that is amenable to parasitological diagnosis—the microfilariae (mf)—are used (e.g., diethylcarbamazine [DEC], ivermectin). Ultrasound to establish killing of adult worms in nodules in onchocerciasis may produce equivocal results [34], and in any case, current annual or semi-annual treatment regimens are not significantly macrofilaricidal. Thus, assessment of “cure” after ivermectin therapy is problematic, and without other markers it hampers study of possible drug resistance. One approach has been to profile the responses of individuals to ivermectin treatment by recording the rates of reappearance of microfilariae in the skin after drug treatment, with a faster than expected rate of repopulation serving as an indicator of suboptimal response [35], [36]. A complementary approach is the histological examination of female worms in nodules removed at approximately 3 months after ivermectin treatment, or the preparation of embryograms from the females' uterine content to assess resumption of microfilarial production [35], [36]. As ivermectin treatment sterilises adult female worms for a period after treatment, after several rounds of ivermectin (>4–6 rounds), the uterus of adult female worms should be free of live, stretched mf 3 months after treatment. Also, if active transmission is still ongoing in an area under prolonged ivermectin control, these methods allow assessment of the presence of young, incoming worms [37].

In LF, the detection of the “filarial dance sign” in worm nests by ultrasound has been advocated to determine adult worm viability after macrofilaricidal chemotherapy (e.g., DEC, anti-*Wolbachia* therapy) [38], [39]. The disappearance of parasite antigen can also serve as a surrogate for cure [40].

### Application of Diagnostics for Disease Mapping, Surveillance, and Mathematical Modelling

Given the imperfections of currently available diagnostics for filariases, particularly with respect to the invasive and insensitive nature of parasitological diagnosis, and the often critical need to undertake cost-effective mapping, a number of novel techniques have been developed to overcome the shortcomings of diagnostics. For onchocerciasis, REMO began with work to identify proximity to blackfly breeding sites and community nodule prevalence [29]. More recently, this has been supplemented by PCR screening of pools of flies [41], and the DEC patch test [42]. A summary of specific issues in diagnostic testing for onchocerciasis is presented in Table 4.

**Table 4:** A Comparison of Current Diagnostic Tests for Human Onchocerciasis.

Test	Specificity	Sensitivity	Interference by *Onchocerca ochengi*	Throughput	Cost	Application
Skin snip	≤100%	Low	No	Low	Low	Field
Nodule palpation	Moderate	Low	No	High	Low	Field
Snip PCR	≤100%	≤100%	No	Low	High	Lab
Scratch PCR	≤100%	≤100%	No	Low	High	Lab
DEC patch	98%	36%–83%	No	Low	Low	Lab
Fly dissection	Low	Low	Yes	Low	Moderate	Field
Fly pool PCR	High	High	No	High	Varies	Lab
Antibody ELISA	≤100%	≤100%	No	High	Mod	Lab

Modified from Boatin et al. [106].

For bancroftian filariasis, CFA assays, in the form of immunochromatographic card tests (ICTs), have been successfully incorporated into programmes to map the distribution of bancroftian filariasis [43], [44], but see the introductory article of this collection for problems with this test [45]. The occurrence of SAEs following ivermectin distribution in areas where *L. loa* infection is highly prevalent [46] has required the development of a model-based geostatistical methodology [47] to map the risk of *L. loa* infection being above a predetermined level (20% prevalence) in areas co-endemic for onchocerciasis. The first approach was based on using elevation and remote sensing images to define high-risk areas based on geographic factors such as vegetation cover and forest boundaries that defined the limits of breeding of the *Chrysops* vector [48]. A second qualitative method, Rapid Assessment Procedure for *Loa loa* (RAPLOA) uses a standardised questionnaire to enquire for history of eye worm or Calabar swelling alone or in conjunction with confirmation of presence of *L. loa* microfilaraemia in a survey sample [49]. The relationship between prevalence of microfilaraemia and prevalence of eyeworm passage has been incorporated into the geostatistical model [50].

## Schistosomiases (including Infections by *Schistosoma mansoni*, *S. haematobium*, and *S. japonicum*)

To date, most schistosomiasis control programmes have aimed at morbidity reduction rather than elimination of infection [51]. MDA with praziquantel is the main tool for morbidity control [52]. As the prevalence of schistosomiasis decreases, the need for improved diagnostic approaches for surveillance purposes will significantly increase, and in some environments where the causative parasite is a zoonosis (*S. japonicum*), increasing attention to the animal reservoirs will be required. A detailed discussion of the strengths and weaknesses of diagnostic tests appropriate for diagnosis of schistosomiasis is available in Text S3, and a summary of current diagnostic tests for schistosomiasis is presented in Table 5. The research priorities for schistosomiasis diagnosis are summarised in Box 3.

**Table 5:** Diagnostics Available for Schistosomiasis.

	Schistosomiasis		
	*Schistosoma mansoni*	*Schistosoma haematobium*	*Schistosoma japonicum*
Parasitological diagnosis	Stool Kato-Katz	Urine filtration	Stool Kato-Katz
Antibody detection	✓	✓	✓
Antigen detection	+	+/−	N/A
PCR	-----Experimental-----		
Assessment of infection intensity	Stool Kato-Katz	Urinary egg count	Stool Kato-Katz
Assessment of drug efficacy	Clearance of eggs from stool	Clearance of eggs from urine	Clearance of eggs from stool
Mapping/elimination	Seroepidemiology	Seroepidemiology	Seroepidemiology

✓, Available or method of choice; N/A, not available; +/−, acceptable but not ideal.

### Infection Intensity

Although estimation of the intensity of schistosomiasis infection is an essential requirement for the implementation of control programmes, M&E, for assessment of efficacy of anti-bilharzial drugs, and as the most important determinant of morbidity, available diagnostics are suboptimal for this purpose.

Quantitative egg counting following urine filtration or Kato-Katz smear are the standard techniques for estimation of intensity of *Schistosoma* infection [53]. However, these methods are confounded by a number of factors, including the overdispersed nature of schistosome egg output in stool, and the daily variation in excretion [54]. It has been recommended that replicate faecal/urine samples over several (ideally a minimum of three) consecutive days be used to quantify infection intensity. This requirement is most important in low endemicity settings, in monitoring of control programmes, and in chemotherapy efficacy trials [55]–[59]. However, such approaches are not logistically or financially feasible except in research settings. Statistical methods have been proposed to improve accuracy in the parasitological estimates [56].

### Application of Diagnostics for Assessment of Response to Anthelmintic Chemotherapy including Detection of Anthelmintic Resistance

A number of reports from endemic areas have suggested that resistance or tolerance to praziquantel might exist in *S. mansoni* in Egypt [60] and Senegal [61], [62], making the need for sensitive diagnostic techniques for M&E of control programmes and praziquantel chemotherapy a priority. Monitoring the efficacy of praziquantel in schistosomiasis has relied on the relatively insensitive method of measuring reduction in egg excretion following treatment [63]. In addition to any variations in drug effect on the worms, several factors can confound the interpretation of such studies. These include variability in pharmacokinetics of praziquantel in different individuals, the effect of differences in immune responses to the worms on drug effect, and the maturity of worms in the human host (praziquantel is relatively ineffective against juvenile worms) [64], [65]. While a number of approaches to overcoming the significant impediments to detecting drug resistance using clinical efficacy as the parameter have been reported, none are readily deployed at programmatic levels. One such approach has been to transfer clinical isolates from human patients into mice for testing [66], [67]. While this method has enabled detailed laboratory study, it is expensive, it requires sophisticated infrastructure, and is not always successful. A number of in vitro tests have been developed for testing praziquantel sensitivity on schistosome parasites at a variety of life cycle stages [68]–[72]. However, to date none of these tests have been standardised.

### Application of Diagnostics for Disease Mapping, Surveillance, and Mathematical Modelling

As schistosomiasis is characterised by focal distribution and large-scale patterns of transmission that are influenced by climatic and environmental conditions, the integrated use of geographical information systems (GIS), remote sensing, and geostatistics has provided new insights into its ecology and epidemiology at a variety of spatial scales [73]. Nevertheless, the microecology of snail and parasite endemicity may not be readily defined without detailed parasitological surveys [74]. The variability in egg output noted above, and its relationship with infection prevalence and intensity, has received considerable attention in the development and validation of mathematical models of schistosomiasis [75], [76]. A positive and statistically significant relationship between serum concentration of circulating cathodic antigen (CCA) and circulating anodic antigen (CAA) (as measures of worm burden) and egg output has been reported [77], and such relationships have been used to confirm density-dependent fecundity in *S. mansoni* [78]. More recently, attention has focused on modelling the impact of chemotherapy on parasite genetic diversity and drug resistance [79]–[81] as discussed in this collection [5].

## Cystic Hydatid Disease

The key interventions for control of cystic hydatid disease (CHD) are continuous mass chemotherapy with praziquantel administered to dogs along with changes to husbandry so that raw offal is not fed to dogs. The development of an efficacious sheep vaccine (EG95) [82] and promising results from early studies on a dog vaccine [83] indicate that these interventions will also become useful tools. There are no standard guidelines for monitoring the effect of control programmes. A detailed discussion of the strengths and weaknesses of diagnostic tests appropriate for diagnosis of cystic hydatid disease is available in Text S4, and the research priorities for CHD diagnosis are summarised in Box 3.

### Infection Intensity

Quantification of the burden of *Echinococcus* worms in dogs can be undertaken by egg counting and by worm counting after arecoline purge. As even a few lightly infected dogs can sustain infection transmission, due to the high biotic potential of cestodes, the intensity of infection is a parameter of less importance in echinococcosis.

### Application of Diagnostics for Assessment of Response to Anthelmintic Chemotherapy including Detection of Anthelmintic Resistance

As described above, the main control tool is mass chemotherapy of dogs; thus, surveillance for treatment efficacy in individual dogs, reinfection rates, or emerging resistance are not priority parameters for surveillance. Instead the number and coverage of dog treatments is the most important variable in control programmes.

### Application of Diagnostics for Disease Mapping, Surveillance, and Mathematical Modelling

The transmission dynamics of *E. granulosus* have been comprehensively studied by Gemmell and co-workers [84]; see also the modelling review in this collection for other mathematical studies of the population biology of CHD [5]. Currently, surveillance is mostly based on infection surveillance at slaughter of infected sheep for liver or lung cysts or, in the case of control programmes targeting dogs, coproantigen or copro PCR assessments.

## Taeniasis/Cysticercosis

Control of taeniasis/cysticercosis due to *Taenia solium* is in a much earlier stage. Pilot intervention studies have been undertaken since 1989 using mass chemotherapy of the human population with praziquantel [85]. In further studies interventions entailing health education and combined mass human and porcine chemotherapy have been investigated. More recently, a large-scale elimination programme was applied in Peru [86] using a series of tools described below. A detailed discussion of the strengths and weaknesses of diagnostic tests appropriate for diagnosis of taeniasis and cysticercosis is available in Text S5, and the research priorities for taeniasis/cysticercosis diagnosis are summarised in Box 3.

### Infection Intensity

In both porcine and human cysticercosis, most infected individuals carry a few parasites, and indeed most (>90%) human tapeworm carriers harbour a single tapeworm, with infections with more than two tapeworms being extremely rare, and therefore unlikely to have implications for transmission control. On the other hand, a high number of brain cysts in a human NCC patient is usually associated with that person carrying a tapeworm, and heavy infections in pigs mark the proximity of a human tapeworm carrier. However, identification of heavily infected humans or pigs requires brain imaging in humans or necropsy of pigs; thus, their use in control is very limited. Tongue-positive pigs may serve as a proxy for heavy cysticercosis infections. Whether transmission of taeniasis in endemic populations is mostly driven by the majority of pigs infected with few cysts (and thus difficult to detect), or by the few pigs with many cysts (potential source of infection to many people if eaten) is an unknown but important question [87]. A diagnostic technique to determine intensity of infection in pigs would therefore prove useful.

### Application of Diagnostics for Assessment of Response to Anthelmintic Chemotherapy including Detection of Anthelmintic Resistance

Intestinal tapeworm infections are usually cured with a single dose of niclosamide. The only way to confirm cure is to identify tapeworm scoleces and proglottids following combined niclosamide and purgative therapy [88], a method that is not widely practiced. Coproantigen follow up was used for this purpose in the Peru elimination programme and found to be very efficient. For cysticercosis, the main way of determining cure is by radiologic imaging to determine that cysts have been killed, a methodology not useful for epidemiologic studies. To date, there is no evidence for anthelminthic resistance in taeniasis.

### Application of Diagnostics for Disease Mapping, Surveillance, and Mathematical Modelling

Since no established control programmes have reached the surveillance stage, no standard methods exist. Surveillance of cysticercosis in pigs has been proposed as a practical, inexpensive, and sensitive method for indirectly assessing human risk, and for monitoring the effectiveness of community-based control programmes [87], [89]. Infection in pigs is more prevalent than human infection, and infection prevalence in pigs is subject to more rapid change due to more rapid replacement of the pig population compared to the human population [90]. Slaughterhouse prevalence statistics are liable to underestimate the prevalence of infection in endemic areas, as many pigs are not processed through the formal slaughterhouse system. Indeed, if pig farmers suspect that their pigs have cysticercosis they may avoid taking them to the slaughterhouse [90]. An alternative approach that has been suggested as a more effective way to assess changes in the intensity of environmental contamination with *T. solium* eggs is the placement of “sentinel” pigs from non-endemic areas in an area under surveillance and their monitoring by periodic serology [91].

Human taeniasis usually has very low prevalence and human cysticercosis may present years after infection, so neither of these represent an appropriate target for active surveillance. Screening of household contacts of cases may be a useful mean of identification and treatment of tapeworm carriers [92], [93].

## Food-Borne Trematodiases

The inadequate state of diagnostic tests for zoonotic trematodes, and particularly *Clonorchis sinensis*, *Opisthorchis viverrini*, *Op. felineus*, *Paragonimus* spp., and other related fluke species, including misdiagnosis of infecting species, underestimation of prevalence, and inaccurate estimates of infection intensity, has been recently reviewed [94]. A detailed discussion of the strengths and weaknesses of diagnostic tests appropriate for diagnosis of food-borne trematode infection is available in Text S6, and the research priorities for food-borne trematode diagnosis are summarised in Box 3.

### Infection Intensity

As for intestinal nematodes, faecal egg counts do not correlate particularly well with the actual worm burden [95]. In an autopsy study aimed to assess the relationship between faecal egg count and worm burden of *Op. viverrini*, worms were found in up to 20% individuals with a negative faecal egg examination [96]. Although a positive correlation was observed between worm burden and faecal egg counts in heavier infections, a density-dependent effect on excretion confounded the relationship.

## Bringing New Helminth Diagnostic Tests to Market

As outlined in this manuscript and accompanying supplemental files, there is a significant literature on prototype diagnostic tests for helminth infections. Once prototype diagnostic devices for helminth infections reach an acceptable level of technical performance, their further development beyond proof of concept requires that a range of significant challenges be overcome. As is the case in the more prominent diseases such as AIDS, tuberculosis (TB), and malaria, the development of critically needed diagnostic tests has assumed lower research priority compared to other areas such as vaccine and drug development. The lack of funding dedicated to development of diagnostics for helminth infections remains a significant impediment, as discussed in Boatin et al. [45] in this collection. This is a reflection of the relatively small funding pool available for helminths overall (see below).

The likely limited interest in commercialisation, a consequence of the limited financial return, is a major impediment to the development of diagnostic tests. This is due to a range of factors including: a) the low priority for making a specific diagnosis of most of these conditions in endemic settings; b) the low royalty for licensing (<5% in most settings) [97]; and c) the significant costs associated with the commercial scale manufacture and marketing of such tests.

Further, the significant investment is required for diagnostic tests to gain regulatory approval from the US Food and Drug Administration (FDA) [98] or the European Union Medical Device Safety Service (MDSS; http://www.mdss.com/IVDD/ivddtoc.htm), and represents a further deterrent. While gaining formal regulatory approval may not be essential for the deployment of a new diagnostic test for helminth infections or other NTDs, such approval carries significant advantages. A minimum requirement for purchase is evidence of good manufacturing practices, as documented by either compliance with ISO 13485:2003 or 21 CFR 820 from the FDA. Once such compliance has been demonstrated, it is likely to be easier to gain financial support from donors and funders for purchase of a diagnostic test for use in public health control programmes.

Despite these challenges, examples exist of diagnostic devices or platforms relevant to helminth infections and others NTDs that have successfully reached market. These include circulating antigen tests for filariasis [99], [100], rapid diagnostic tests for malaria [101], the k39 antibody detection test for leishmaniasis [102], and the urinary CCA test for schistosomiasis [103]. Not well documented in the scientific literature are the difficulties that each of these platforms experienced in reaching market and then maintaining a market presence. Of these, only a malaria rapid diagnostic test from a single manufacturer (BinaxNOW Malaria test kit; Inverness, Scarborough, Maine, United States) has gained FDA approval as a diagnostic device.

Examination of the landscape of diagnostic tests and their pathway to deployment therefore indicates that the standard commercial pathway is not well suited to meeting the needs for diagnostics to aid in the control of helminth parasites. Indeed, in a recent review of the global investment for NTDs [104], the sum of funding for research and development (R&D) for diagnostic tests for helminth diseases was $US1.4 million, a figure that represented 1.8% of total R&D funding for helminths (Table 6). While this relative lack of investment is by no means unique to these pathogens, it is of major importance in consideration of the need to develop and deploy much-needed new diagnostic tests for helminth infections. Indeed, it has become clear that to reach the targets of the Millennium Development Goals (MDGs) for many of the priority infectious diseases such as TB, there is a major and unmet need for the development of diagnostic tests.

**Table 6:** Funding of R&D Products for Helminthiases in 2009 (in US$ Million).

Helminth Infection	Basic Research	Drugs	Vaccines	Vectors/Snails	Diagnostics	Unspecified	Total	%
Hookworm infection	2.7	–	6.9	–	–	–	9.7	12.2
Ascariasis	2.1	–	–	–	–	–	2.1	2.6
Trichuriasis	1.0	0.035	–	–	–	–	1.0	1.3
Strongyloidiasis & others	1.4	0.035	–	–	0.142	–	1.5	1.9
Lymphatic filariasis	4.9	5.0		1.3	–	3.6	14.9	18.8
Onchocerciasis	1.9	8.9	0.807	?	0.163	1.3	13.0	16.5
Schistosomiasis	16.1	0.642	2.0	–	0.983	2.0	21.9	27.6
Taeniasis	2.3	–	Unknown	0.535	–	–	2.9	3.7
Multiple helminthiases	9.1	0.582	–	–	0.103	2.3	12.1	15.3
Total	41.7	15.3	9.9	1.9	1.4	9.3	79.4	
%	52.5	19.3	12.4	2.3	1.8	11.7		

Adapted from [104].

A novel approach to bridging the gap between demonstrating a technically satisfactory diagnostic assay, and bringing the product in question to the market entails the formation of organisations specifically tasked with this mission. The foremost example of such a Product Development and Implementation Partnership (PDIP) is the Foundation for Innovative New Diagnostics (FIND; http://www.finddiagnostics.org), an organisation that focuses on a small number of diseases including TB, sleeping sickness, and malaria. It is supported by the World Health Organization, the Bill & Melinda Gates Foundation, the European Commission, the Government of The Netherlands, UNITAID, DFID, and others.

As outlined by Murdoch in a FIND monograph [105], a range of complex issues need to be dealt with to successfully develop such diagnostics. These include: a) identifying specific patients' needs in developing countries and targeting products to all the levels of the health system; b) understanding the market—potential volumes, distribution of products, and end user profiles; c) arranging the financial considerations to ensure they cover both research and development costs; d) settling issues regarding intellectual property rights for products with no or limited financial return; e) finding the right manufacturing approach and harnessing possibilities for technology transfer; f) creating methods for evaluating the product through clinical trials and for introducing it at country level; g) working with governments to adapt national policies and approaches; h) working with local laboratories to ensure they have the capacity to use the products effectively; and i) working with donors and national governments to make sure tests can be purchased at prices affordable for all.

It is apparent that for the successful development of the novel diagnostic tests required for the control of helminth parasites, similar approaches will be required. However, even if such a model for development of diagnostic tests were implemented, a system would need to be put in place to finance procurement, as requiring the end user to pay will likely result in tests not being used.

## Concluding Remarks

Although a significant number of obstacles of a technical nature impede the development of diagnostics appropriate to support the ambitious programmes now in place to control the NTDs in general, and the human helminthiases in particular, there is good evidence that the tools are available to overcome these obstacles. Whether the patchwork of activities as currently being undertaken largely by enthusiastic researchers, for the most part working in academic settings, will result in the expeditious development of these much needed tests is uncertain. As identified in the G-Finder report [104], the expenditure on R&D for NTD diagnostics represents only a small proportion of the funding expended in these areas, and is yet an even smaller proportion of the operational costs of undertaking the comprehensive parasite control programmes that are currently being advocated or implemented [3], [24]. Many lessons can be learned from the initiative to improve the development and public health deployment of rapid diagnostics for malaria, led by TDR and FIND. Although funding for assay development is needed, the largest needs relate to programmatic leadership and support to bridge the gap between the demonstration of satisfactory technical performance of diagnostic platforms in field or clinical settings, and their deployment in large-scale public health programmes for the control and elimination of human helminthiases.

## Supporting Information

Text S1 Diagnostics for Soil-Transmitted Helminthiases.
(DOCX)

Text S2 Diagnostics for Filariases.
(DOCX)

Text S3 Diagnostics for Schistosomiasis.
(DOCX)

Text S4 Diagnostics for Cystic Hydatid Disease.
(DOCX)

Text S5 Diagnostics for Taeniasis Cysticercosis.
(DOCX)

Text S6 Diagnostics for Food-Borne Trematodiases.
(DOCX)

## References

[1] Lustigman S, Prichard RK, Gazzinelli A, Grant WN, Boatin BA (2012). A research agenda for helminth diseases of humans: the problem of helminthiases.. PLoS Negl Trop Dis.

[2] Vercruysse J, Albonico M, Behnke J, Kotze, Prichard R (2011). Is anthelmintic resistance a concern for the control of human soil-transmitted helminths?. Int J Parasitol: Drugs and Drug Resistance.

[3] Hotez PJ, Molyneux DH, Fenwick A, Kumaresan J, Sachs SE (2007). Control of neglected tropical diseases.. N Engl J Med.

[4] Levecke B, Behnke JM, Ajjampur SS, Albonico M, Ame SM (2011). A comparison of the sensitivity and fecal egg counts of the McMaster egg counting and Kato-Katz thick smear methods for soil-transmitted helminths.. PLoS Negl Trop Dis.

[5] Basáñez MG, McCarthy JS, French MD, Yang GJ, Walker M (2012). A research agenda for helminth diseases of humans: modelling for control and elimination.. PLoS Negl Trop Dis.

[6] Geary TG, Woo K, McCarthy JS, Mackenzie CD, Horton J (2010). Unresolved issues in anthelmintic pharmacology for helminthiases of humans.. Int J Parasitol.

[7] Katz N, Chaves A, Pellegrino J (1972). A simple device for quantitative stool thick-smear technique in Schistosomiasis mansoni.. Rev Inst Med Trop Sao Paulo.

[8] Goodman D, Haji HJ, Bickle QD, Stoltzfus RJ, Tielsch JM (2007). A comparison of methods for detecting the eggs of *Ascaris*, *Trichuris*, and hookworm in infant stool, and the epidemiology of infection in Zanzibari infants.. Am J Trop Med Hyg.

[9] Ramsan M, Montresor A, Foum A, Ameri H, Di Matteo L (1999). Independent evaluation of the Nigrosin-Eosin modification of the Kato-Katz technique.. Trop Med Int Health.

[10] Dacombe RJ, Crampin AC, Floyd S, Randall A, Ndhlovu R (2007). Time delays between patient and laboratory selectively affect accuracy of helminth diagnosis.. Trans R Soc Trop Med Hyg.

[11] Cringoli G (2006). FLOTAC, a novel apparatus for a multivalent faecal egg count technique.. Parassitologia.

[12] Knopp S, Glinz D, Rinaldi L, Mohammed KA, N'Goran EK (2009). FLOTAC: a promising technique for detecting helminth eggs in human faeces.. Trans R Soc Trop Med Hyg.

[13] Utzinger J, Rinaldi L, Lohourignon LK, Rohner F, Zimmermann MB (2008). FLOTAC: a new sensitive technique for the diagnosis of hookworm infections in humans.. Trans R Soc Trop Med Hyg.

[14] Hall A, Holland C (2000). Geographical variation in *Ascaris lumbricoides* fecundity and its implications for helminth control.. Parasitol Today.

[15] Walker M, Hall A, Anderson RM, Basáñez MG (2009). Density-dependent effects on the weight of female *Ascaris lumbricoides* infections of humans and its impact on patterns of egg production.. Parasit Vectors.

[16] Kotze AC, Kopp SR (2008). The potential impact of density dependent fecundity on the use of the faecal egg count reduction test for detecting drug resistance in human hookworms.. PLoS Negl Trop Dis.

[17] Bennett A, Guyatt H (2000). Reducing intestinal nematode infection: efficacy of albendazole and mebendazole.. Parasitol Today.

[18] Keiser J, Utzinger J (2008). Efficacy of current drugs against soil-transmitted helminth infections: systematic review and meta-analysis.. JAMA.

[19] Coles GC, Jackson F, Pomroy WE, Prichard RK, von Samson-Himmelstjerna G (2006). The detection of anthelmintic resistance in nematodes of veterinary importance.. Vet Parasitol.

[20] Albonico M, Wright V, Ramsan M, Haji HJ, Taylor M (2005). Development of the egg hatch assay for detection of anthelminthic resistance in human hookworms.. Int J Parasitol.

[21] De Clercq D, Sacko M, Behnke J, Gilbert F, Dorny P (1997). Failure of mebendazole in treatment of human hookworm infections in the southern region of Mali.. Am J Trop Med Hyg.

[22] Kotze AC, Coleman GT, Mai A, McCarthy JS (2005). Field evaluation of anthelmintic drug sensitivity using in vitro egg hatch and larval motility assays with *Necator americanus* recovered from human clinical isolates.. Int J Parasitol.

[23] Kotze AC, Lowe A, O'Grady J, Kopp SR, Behnke JM (2009). Dose-response assay templates for in vitro assessment of resistance to benzimidazole and nicotinic acetylcholine receptor agonist drugs in human hookworms.. Am J Trop Med Hyg.

[24] Prichard RK, Basáñez MG, Boatin BA, McCarthy JS, García HH (2012). A research agenda for helminth diseases of humans: intervention for control and elimination.. PLoS Negl Trop Dis.

[25] Diawara A, Drake LJ, Suswillo RR, Kihara J, Bundy DAP (2009). Assays to detect beta-tubulin codon 200 polymorphism in *Trichuris trichiura* and *Ascaris lumbricoides*.. PLoS Negl Trop Dis.

[26] Schwenkenbecher JM, Albonico M, Bickle Q, Kaplan RM (2007). Characterization of beta-tubulin genes in hookworms and investigation of resistance-associated mutations using real-time PCR.. Mol Biochem Parasitol.

[27] Albonico M, Wright V, Bickle Q (2004). Molecular analysis of the beta-tubulin gene of human hookworms as a basis for possible benzimidazole resistance on Pemba Island.. Mol Biochem Parasitol.

[28] Bennett AB, Anderson TJ, Barker GC, Michael E, Bundy DA (2002). Sequence variation in the *Trichuris trichiura* beta-tubulin locus: implications for the development of benzimidazole resistance.. Int J Parasitol.

[29] Ngoumou P, Walsh JF, Mace JM (1994). A rapid mapping technique for the prevalence and distribution of onchocerciasis: a Cameroon case study.. Ann Trop Med Parasitol.

[30] Duerr HP, Raddatz G, Eichner M (2008). Diagnostic value of nodule palpation in onchocerciasis.. Trans R Soc Trop Med Hyg.

[31] Weil GJ, Blair LS, Ewanciw DV, Malatesta PF (1986). Use of parasite antigen detection to monitor the success of drug therapy in *Dirofilaria immitis*-infected dogs.. J Parasitol.

[32] Hussein O, El Setouhy M, Ahmed ES, Kandil AM, Ramzy RM (2004). Duplex Doppler sonographic assessment of the effects of diethylcarbamazine and albendazole therapy on adult filarial worms and adjacent host tissues in Bancroftian filariasis.. Am J Trop Med Hyg.

[33] Awadzi K, Edwards G, Opoku NO, Ardrey AE, Favager S (2004). The safety, tolerability and pharmacokinetics of levamisole alone, levamisole plus ivermectin, and levamisole plus albendazole, and their efficacy against *Onchocerca volvulus*.. Ann Trop Med Parasitol.

[34] Mand S, Marfo-Debrekyei Y, Debrah A, Buettner M, Batsa L (2005). Frequent detection of worm movements in onchocercal nodules by ultrasonography.. Filaria J.

[35] Awadzi K, Attah SK, Addy ET, Opoku NO, Quartey BT (2004). Thirty-month follow-up of sub-optimal responders to multiple treatments with ivermectin, in two onchocerciasis-endemic foci in Ghana.. Ann Trop Med Parasitol.

[36] Awadzi K, Boakye DA, Edwards G, Opoku NO, Attah SK (2004). An investigation of persistent microfilaridermias despite multiple treatments with ivermectin, in two onchocerciasis-endemic foci in Ghana.. Ann Trop Med Parasitol.

[37] Specht S, Brattig N, Buttner M, Buttner DW (2009). Criteria for the differentiation between young and old *Onchocerca volvulus* filariae.. Parasitol Res.

[38] Noroes J, Dreyer G, Santos A, Mendes VG, Medeiros Z (1997). Assessment of the efficacy of diethylcarbamazine on adult *Wuchereria bancrofti in vivo*.. Trans R Soc Trop Med Hyg.

[39] Dreyer G, Noroes J, Amaral F, Nen A, Medeiros Z (1995). Direct assessment of the adulticidal efficacy of a single dose of ivermectin in bancroftian filariasis.. Trans R Soc Trop Med Hyg.

[40] McCarthy JS, Guinea A, Weil GJ, Ottesen EA (1995). Clearance of circulating filarial antigen as a measure of the macrofilaricidal activity of diethylcarbamazine in *Wuchereria bancrofti* infection.. J Infect Dis.

[41] Boatin BA, Richards FO (2006). Control of onchocerciasis.. Adv Parasitol.

[42] Ozoh G, Boussinesq M, Bissek AC, Kobangue L, Kombila M (2007). Evaluation of the diethylcarbamazine patch to evaluate onchocerciasis endemicity in Central Africa.. Trop Med Int Health.

[43] Gyapong JO, Kyelem D, Kleinschmidt I, Agbo K, Ahouandogbo F (2002). The use of spatial analysis in mapping the distribution of bancroftian filariasis in four West African countries.. Ann Trop Med Parasitol.

[44] Onapa AW, Simonsen PE, Baehr I, Pedersen EM (2005). Rapid assessment of the geographical distribution of lymphatic filariasis in Uganda, by screening of schoolchildren for circulating filarial antigens.. Ann Trop Med Parasitol.

[45] Boatin BA, Basáñez MG, Prichard RK, Awadzi K, Barakat RM (2012). A research agenda for helminth diseases of humans: towards control and elimination.. PLoS Negl Trop Dis.

[46] Gardon J, Gardon-Wendel N, Demanga-Ngangue, Kamgno J, Chippaux JP (1997). Serious reactions after mass treatment of onchocerciasis with ivermectin in an area endemic for *Loa loa* infection.. Lancet.

[47] Diggle P, Tawn J, Moyeed RA (1998). Model-based geostatistics.. J R Stat Soc Ser C (Appl Stat).

[48] Thomson MC, Obsomer V, Dunne M, Connor SJ, Molyneux DH (2000). Satellite mapping of *Loa loa* prevalence in relation to ivermectin use in west and central Africa.. Lancet.

[49] Takougang I, Meremikwu M, Wandji S, Yenshu EV, Aripko B (2002). Rapid assessment method for prevalence and intensity of *Loa loa* infection.. Bull World Health Organ.

[50] Crainiceanu C, Diggle P, Rowlingson B (2008). Bivariate binomial spatial modeling of *Loa loa* prevalence in tropical Africa.. J Am Stat Assoc.

[51] WHO (1985). The control of schistosomiasis. Report of a WHO Expert Committee.. World Health Organ Tech Rep Ser.

[52] WHO (2002). Prevention and control of schistosomiasis and soil-transmitted helminthiasis.. World Health Organ Tech Rep Ser.

[53] Feldmeier H, Jordan P, Webbe G, Sturrock R (1993). Diagnosis in human schistosomiasis.. Human schistosomiasis.

[54] Yu JM, de Vlas SJ, Yuan HC, Gryseels B (1998). Variations in fecal *Schistosomiasis japonicum* egg counts.. Am J Trop Med Hyg.

[55] Berhe N, Medhin G, Erko B, Smith T, Gedamu S (2004). Variations in helminth faecal egg counts in Kato-Katz thick smears and their implications in assessing infection status with *Schistosoma mansoni*.. Acta Trop.

[56] Booth M, Vounatsou P, N'Goran EK, Tanner M, Utzinger J (2003). The influence of sampling effort and the performance of the Kato-Katz technique in diagnosing *Schistosoma mansoni* and hookworm co-infections in rural Cote d'Ivoire.. Parasitology.

[57] Ebrahim A, El-Morshedy H, Omer E, El-Daly S, Barakat RM (1997). Evaluation of the Kato-Katz thick smear and formol ether sedimentation techniques for quantitative diagnosis of *Schistosoma mansoni* infection.. Am J Trop Med Hyg.

[58] Kongs A, Marks G, Verle P, Van der Stuyft P (2001). The unreliability of the Kato-Katz technique limits its usefulness for evaluating *S. mansoni* infections.. Trop Med Int Health.

[59] Utzinger J, Booth M, N'Goran EK, Muller I, Tanner M (2001). Relative contribution of day-to-day and intra-specimen variation in faecal egg counts of *Schistosoma mansoni* before and after treatment with praziquantel.. Parasitology.

[60] Ismail M, Metwally A, Farghaly A, Bruce J, Tao LF (1996). Characterization of isolates of *Schistosoma mansoni* from Egyptian villagers that tolerate high doses of praziquantel.. Am J Trop Med Hyg.

[61] Gryseels B, Stelma FF, Talla I, van Dam GJ, Polman K (1994). Epidemiology, immunology and chemotherapy of *Schistosoma mansoni* infections in a recently exposed community in Senegal.. Trop Geogr Med.

[62] Stelma FF, Talla I, Verle P, Niang M, Gryseels B (1994). Morbidity due to heavy *Schistosoma mansoni* infections in a recently established focus in northern Senegal.. Am J Trop Med Hyg.

[63] Doenhoff MJ (1998). Is Schistosomicidal Chemotherapy Sub-curative? Implications for Drug Resistance.. Parasitol Today.

[64] Botros S, Sayed H, Amer N, El-Ghannam M, Bennett JL (2005). Current status of sensitivity to praziquantel in a focus of potential drug resistance in Egypt.. Int J Parasitol.

[65] Erhart A, Dorny P, Van De N, Vien HV, Thach DC (2002). Taenia solium cysticercosis in a village in northern Viet Nam: seroprevalence study using an ELISA for detecting circulating antigen.. Trans R Soc Trop Med Hyg.

[66] Cioli D, Botros SS, Wheatcroft-Francklow K, Mbaye A, Southgate V (2004). Determination of ED50 values for praziquantel in praziquantel-resistant and -susceptible *Schistosoma mansoni* isolates.. Int J Parasitol.

[67] Melman SD, Steinauer ML, Cunningham C, Kubatko LS, Mwangi IN (2009). Reduced susceptibility to praziquantel among naturally occurring Kenyan isolates of *Schistosoma mansoni*.. PLoS Negl Trop Dis.

[68] Liang YS, Coles GC, Doenhoff MJ, Southgate VR (2001). In vitro responses of praziquantel-resistant and -susceptible *Schistosoma mansoni* to praziquantel.. Int J Parasitol.

[69] Pica-Mattoccia L, Cioli D (2004). Sex- and stage-related sensitivity of *Schistosoma mansoni* to *in vivo* and *in vitro* praziquantel treatment.. Int J Parasitol.

[70] William S, Botros S (2004). Validation of sensitivity to praziquantel using *Schistosoma mansoni* worm muscle tension and Ca2+-uptake as possible in vitro correlates to in vivo ED50 determination.. Int J Parasitol.

[71] Smout MJ, Kotze AC, McCarthy JS, Loukas A (2010). A novel high throughput assay for anthelmintic drug screening and resistance diagnosis by real-time monitoring of parasite motility.. PLoS Negl Trop Dis.

[72] Lamberton PH, Hogan SC, Kabatereine NB, Fenwick A, Webster JP (2010). In vitro praziquantel test capable of detecting reduced in vivo efficacy in *Schistosoma mansoni* human infections.. Am J Trop Med Hyg.

[73] Brooker S, Alexander N, Geiger S, Moyeed RA, Stander J (2006). Contrasting patterns in the small-scale heterogeneity of human helminth infections in urban and rural environments in Brazil.. Int J Parasitol.

[74] Stothard JR (2009). Improving control of African schistosomiasis: towards effective use of rapid diagnostic tests within an appropriate disease surveillance model.. Trans R Soc Trop Med Hyg.

[75] De Vlas SJ, Gryseels B, Van Oortmarssen GJ, Polderman AM, Habbema JD (1992). A model for variations in single and repeated egg counts in *Schistosoma mansoni* infections.. Parasitology.

[76] De Vlas SJ, Engels D, Rabello AL, Oostburg BF, Van Lieshout L (1997). Validation of a chart to estimate true *Schistosoma mansoni* prevalences from simple egg counts.. Parasitology.

[77] Van Lieshout L, Polderman AM, De Vlas SJ, De Caluwe P, Krijger FW (1995). Analysis of worm burden variation in human *Schistosoma mansoni* infections by determination of serum levels of circulating anodic antigen and circulating cathodic antigen.. J Infect Dis.

[78] Polman K, De Vlas SJ, Van Lieshout L, Deelder AM, Gryseels B (2001). Evaluation of density-dependent fecundity in human *Schistosoma mansoni* infections by relating egg counts to circulating antigens through Deming regression.. Parasitology.

[79] Castillo-Chavez C, Feng Z, Xu D (2008). A schistosomiasis model with mating structure and time delay.. Math Biosci.

[80] Feng Z, Curtis J, Minchella DJ (2001). The influence of drug treatment on the maintenance of schistosome genetic diversity.. J Math Biol.

[81] Xu D, Curtis J, Feng Z, Minchella DJ (2005). On the role of schistosome mating structure in the maintenance of drug resistant strains.. Bull Math Biol.

[82] Lightowlers MW, Jensen O, Fernandez E, Iriarte JA, Woollard DJ (1999). Vaccination trials in Australia and Argentina confirm the effectiveness of the EG95 hydatid vaccine in sheep.. Int J Parasitol.

[83] Zhang W, Zhang Z, Shi B, Li J, You H (2006). Vaccination of dogs against *Echinococcus granulosus*, the cause of cystic hydatid disease in humans.. J Infect Dis.

[84] Gemmell MA, Lawson JR, Roberts MG (1986). Population dynamics in echinococcosis and cysticercosis: biological parameters of *Echinococcus granulosus* in dogs and sheep.. Parasitology.

[85] Cruz M, Davis A, Dixon H, Pawlowski ZS, Proano J (1989). Operational studies on the control of *Taenia solium* taeniasis/cysticercosis in Ecuador.. Bull World Health Organ.

[86] Mahanty S, Garcia HH (2010). Cysticercosis and neurocysticercosis as pathogens affecting the nervous system.. Prog Neurobiol.

[87] Willingham AL, Engels D (2006). Control of Taenia solium cysticercosis/taeniosis.. Adv Parasitol.

[88] Jeri C, Gilman RH, Lescano AG, Mayta H, Ramirez ME (2004). Species identification after treatment for human taeniasis.. Lancet.

[89] Gonzalez LM, Montero E, Puente S, Lopez-Velez R, Hernandez M (2002). PCR tools for the differential diagnosis of *Taenia saginata* and *Taenia solium* taeniasis/cysticercosis from different geographical locations.. Diagn Microbiol Infect Dis.

[90] Gonzalez AE, Garcia HH, Gilman RH, Tsang VC (2003). Control of *Taenia solium*.. Acta Trop.

[91] Sarti E, Flisser A, Schantz PM, Gleizer M, Loya M (1997). Development and evaluation of a health education intervention against *Taenia solium* in a rural community in Mexico.. Am J Trop Med Hyg.

[92] Garcia HH, Gilman RH, Gonzalez AE, Pacheco R, Verastegui M (1999). Human and porcine *Taenia solium* infection in a village in the highlands of Cusco, Peru. The Cysticercosis Working Group in Peru.. Acta Trop.

[93] Sarti E, Schantz PM, Plancarte A, Wilson M, Gutierrez IO (1992). Prevalence and risk factors for *Taenia solium* taeniasis and cysticercosis in humans and pigs in a village in Morelos, Mexico.. Am J Trop Med Hyg.

[94] Johansen MV, Sithithaworn P, Bergquist R, Utzinger J (2010). Towards improved diagnosis of zoonotic trematode infections in Southeast Asia.. Adv Parasitol.

[95] Dorny P, Praet N, Deckers N, Gabriel S (2009). Emerging food-borne parasites.. Vet Parasitol.

[96] Sithithaworn P, Tesana S, Pipitgool V, Kaewkes S, Pairojkul C (1991). Relationship between faecal egg count and worm burden of *Opisthorchis viverrini* in human autopsy cases.. Parasitology.

[97] Adams AF, Calcagno PT, Naydenova B (2008). Patent royalty rates: a look at recent court decisions.. http://www.integrityrehab.com/Patent%20Royalty%20Rates%20-%20Recent%20Court%20Decisions%20-%20Adams%204-20-08.pdf.

[98] Mansfield E, O'Leary TJ, Gutman SI (2005). Food and Drug Administration regulation of in vitro diagnostic devices.. J Mol Diagn.

[99] More SJ, Copeman DB (1990). A highly specific and sensitive monoclonal antibody-based ELISA for the detection of circulating antigen in bancroftian filariasis.. Trop Med Parasitol.

[100] Weil GJ, Jain DC, Santhanam S, Malhotra A, Kumar H (1987). A monoclonal antibody-based enzyme immunoassay for detecting parasite antigenemia in bancroftian filariasis.. J Infect Dis.

[101] Bell D, Wongsrichanalai C, Barnwell JW (2006). Ensuring quality and access for malaria diagnosis: how can it be achieved?. Nat Rev Microbiol.

[102] Chappuis F, Rijal S, Soto A, Menten J, Boelaert M (2006). A meta-analysis of the diagnostic performance of the direct agglutination test and rK39 dipstick for visceral leishmaniasis.. BMJ.

[103] van Dam GJ, Wichers JH, Ferreira TM, Ghati D, van Amerongen A (2004). Diagnosis of schistosomiasis by reagent strip test for detection of circulating cathodic antigen.. J Clin Microbiol.

[104] Moran M, Guzman J, Henderson K, Ropars A, McDonald A (2009). Neglected disease research & development: new times, new trends.

[105] Murdoch T (2008). The diagnostic path to a better world.

[106] Boatin BA, Toe L, Alley ES, Nagelkerke NJ, Borsboom G (2002). Detection of *Onchocerca volvulus* infection in low prevalence areas: a comparison of three diagnostic methods.. Parasitology.

[107] Lipner EM, Dembele N, Souleymane S, Alley WS, Prevots DR (2006). Field applicability of a rapid-format anti-Ov-16 antibody test for the assessment of onchocerciasis control measures in regions of endemicity.. J Infect Dis.

[108] Chambers EW, McClintock SK, Avery MF, King JD, Bradley MH (2009). Xenomonitoring of *Wuchereria bancrofti* and Dirofilaria immitis infections in mosquitoes from American Samoa: trapping considerations and a comparison of polymerase chain reaction assays with dissection.. Am J Trop Med Hyg.

[109] Lammie PJ, Weil G, Noordin R, Kaliraj P, Steel C (2004). Recombinant antigen-based antibody assays for the diagnosis and surveillance of lymphatic filariasis - a multicenter trial.. Filaria J.

[110] Mladonicky JM, King JD, Liang JL, Chambers E, Pa'au M (2009). Assessing transmission of lymphatic filariasis using parasitologic, serologic, and entomologic tools after mass drug administration in American Samoa.. Am J Trop Med Hyg.

[111] WHO (2008). Elimination of schistosomiasis from low-transmission areas: report of a WHO informal consultation..

[112] Zhu YC (2005). Immunodiagnosis and its role in schistosomiasis control in China: a review.. Acta Trop.

[113] Lier T, Johansen MV, Hjelmevoll SO, Vennervald BJ, Simonsen GS (2008). Real-time PCR for detection of low intensity *Schistosomiasis japonicum* infections in a pig model.. Acta Trop.

[114] Lier T, Simonsen GS, Wang T, Lu D, Haukland HH (2009). Real-time polymerase chain reaction for detection of low-intensity *Schistosomiasis japonicum* infections in China.. Am J Trop Med Hyg.

