# Supplementary material for: A Research Agenda for Helminth Diseases of Humans: Diagnostics for Control and Elimination Programmes
Source: PLoS Negl Trop Dis. 2012 Apr 24;6(4):e1601. doi: 10.1371/journal.pntd.0001601 (PMC3335877; doi:10.1371/journal.pntd.0001601)
Supplement: Text S1 — Diagnostics for Soil-Transmitted Helminthiases. (DOCX) [file pntd.0001601.s001.docx]

**Text S1. Diagnostics for Soil-Transmitted Helminthiases**

***Parasitological Diagnosis***

Infection with soil-transmitted helminthiases (STHs) can be readily diagnosed by detection of helminth eggs in stool samples using microscopic techniques. Such techniques are generally sufficiently sensitive provided that appropriate methodologies are used (see below). The addition of a concentration step generally improves sensitivity albeit at the price of reducing the ability to assess infection intensity. Specific problems pertain to hookworm and *Strongyloides stercoralis*. Hookworm eggs may lyse if samples are not examined within thirty minutes of preparation of a Kato-Katz slide, and coproculture using the agar plate method, as well as the examination of multiple stool specimens, are required for diagnosis of *St. stercoralis* infection. Likewise coproculture, using the Harada Mori technique is required to differentiate *Ancylostoma duodenale* from *Necator americanus* by microscopic examination of the mouthparts of hookworm larvae.

***Antibody Tests***

Except for the case of strongyloidiasis, the use of antibody tests to detect specific antibody responses to STHs has not been considered necessary for diagnostic purposes, and relatively little work has been undertaken to develop and standardize serological assays for hookworm infection, ascariasis or trichuriasis. For strongyloidiasis, a significant amount of work has been undertaken to develop antibody ELISA diagnostic tests, using both crude larval antigen and recombinant proteins [[1](#_ENREF_1)].

***Antigen Detection***

Some work has been undertaken in the zoonotic hookworm species *An. ceylanicum*, and in *Strongyloides* spp. demonstrating the presence of coproantigen in faeces [[2](#_ENREF_2),[3](#_ENREF_3)]. However, no assay has reached a mature stage of development.

***Molecular Diagnosis***

PCR-based diagnosis of hookworm infection has been developed and subject to pilot testing in human populations [[4-6](#_ENREF_4)]. A multiplex PCR assay has been described and shows promise for quantifying egg counts for hookworm infection and ascariasis [[7](#_ENREF_7),[8](#_ENREF_8)]. Such assays promise the ability to undertake multiplexed and quantitative assessment of STHs.

**References**

1. Siddiqui AA, Berk SL (2001) Diagnosis of *Strongyloides stercoralis* infection.
Clin Infect Dis 33: 1040-1047.

2. Bungiro RD, Jr., Cappello M (2005) Detection of excretory/secretory coproantigens in experimental hookworm infection. Am J Trop Med Hyg 73: 915-920.

3. Sykes AM, McCarthy JS (2011) A coproantigen diagnostic test for *Strongyloides infection*. PLoS Negl Trop Dis 5: e955.

4. Verweij JJ, Brienen EA, Ziem J, Yelifari L, Polderman AM, et al. (2007) Simultaneous detection and quantification of *Ancylostoma duodenale, Necator americanus,* and *Oesophagostomum bifurcum* in fecal samples using multiplex real-time PCR. Am J Trop Med Hyg 77: 685-690.

5. de Gruijter JM, van Lieshout L, Gasser RB, Verweij JJ, Brienen EA, et al. (2005) Polymerase chain reaction-based differential diagnosis of *Ancylostoma duodenale* and *Necator americanus* infections in humans in northern Ghana.
Trop Med Int Health 10: 574-580.

6. Verweij JJ, Pit DS, van Lieshout L, Baeta SM, Dery GD, et al. (2001) Determining the prevalence of *Oesophagostomum bifurcum* and *Necator americanus* infections using specific PCR amplification of DNA from faecal samples.
Trop Med Int Health 6: 726-731.

7. Leles D, Araujo A, Vicente AC, Iniguez AM (2009) Molecular diagnosis of ascariasis from human feces and description of a new Ascaris sp. genotype in Brazil.
Vet Parasitol 163: 167-170.

8. Pecson BM, Barrios JA, Johnson DR, Nelson KL (2006) A real-time PCR method for quantifying viable ascaris eggs using the first internally transcribed spacer region of ribosomal DNA. Appl Environ Microbiol 72: 7864-7872.
