# Supplementary material for: A Research Agenda for Helminth Diseases of Humans: Diagnostics for Control and Elimination Programmes
Source: PLoS Negl Trop Dis. 2012 Apr 24;6(4):e1601. doi: 10.1371/journal.pntd.0001601 (PMC3335877; doi:10.1371/journal.pntd.0001601)
Supplement: Text S2 — Diagnostics for Filariases. (DOCX) [file pntd.0001601.s002.docx]

**Text S2. Diagnostics for Filariasis**

***Parasitological Diagnosis***

Detection of Microfilariae

The filarial parasites (*Onchocerca volvulus*, *Wuchereria bancrofti,* *Brugia malayi* and *Loa loa*) are amenable to parasitological diagnosis by detection of first stage, embryonic (microfilariae or L_1_) larvae. Microfilariae (mf) are found in the skin in *O. volvulus* infection, and the blood in the other three. (The microfilariae of other filarial parasites such as *Mansonella* (=*Dipetalonema*) *perstans* and *M.ozzardi* are also present in blood; those of *Dipetalonema streptocerca* are present in skin, and in mixed infections with the species included in this article, they could present diagnostic difficulties, However, these filarial nematodes are beyond the scope of this review.) For diagnosis of onchocerciasis, the collection and examination of skin snips is a well standardized, albeit suboptimal test. It is prone to low sensitivity in light infection when mf tend to be more aggregated in host skin. Factors influencing the sensitivity of the test include the anatomic sites from where skin snips are taken, the number of snips taken, the duration of snip incubation, and the composition of the medium in which snips are incubated [[1](#_ENREF_1),[2](#_ENREF_2)]. This technique can only detect by inference the presence of fertile adult females and not early (pre-patent) infection. Because taking a skin snip can cause transient pain or discomfort, subjects can become reluctant to submit to sampling, pointing to the necessity to develop a less invasive test. The DEC patch test, a test that works by inducing a local inflammatory response to microfilarial death in the skin immediately below the patch represents an alternative approach. In this test diethylcarbamazine (DEC) is applied to a patch of skin, eliciting a localized, so-called Mazzotti reaction [[3](#_ENREF_3)]. It has been shown to be useful where prevalence of infection is being studied, as it is quicker to perform and avoids invasive sample collection [[4](#_ENREF_4)]. For detection of mf in blood in LF and loaisis, the timing of blood collection should coincide with the time of peak microfilaraemia. Although this depends on the periodicity of the mf in the peripheral circulation, it is most commonly in the middle of the night for nocturnally periodic LF, a time when blood sampling is generally very inconvenient. The administration of DEC in the daytime in areas where mf of *W. bancrofti* and *B. malayi* exhibit nocturnal periodicity provokes mf to enter the peripheral blood [[5](#_ENREF_5)]. Daytime blood sampling is satisfactory for *L. loa*.

Detection of Macrofilariae

The adult worms (macrofilariae) of *O. volvulus* can be detected by palpation of onchocercal nodules (known as onchocercomata). However, nodule palpation is relatively insensitive, not quantitative and non-specific, particularly in areas of low endemicity [[6](#_ENREF_6)]. Sensitivity is hampered by the fact that up to one half of nodules may be located in deep tissues, while specificity is hampered by the fact that other conditions may cause subcutaneous nodules, such as cysticercercosis [[7](#_ENREF_7)], enlarged lymph nodes, etc. Ultrasound examinations of nodules, to confirm the presence of living adult *O. volvulus* may serve as a tool for longitudinal observation *in vivo* of patients undergoing treatment, and as an adjunct to histological evaluation of viability in control programmes [[8](#_ENREF_8)]. For LF, ultrasound examination of afferent lymphatics close to lymph nodes [[9](#_ENREF_9)], typically in the scrotum of adult males can be used to detect worm nests.

***Antibody Tests***

Antibody tests are widely used for serodiagnosis of filarial infections, and have assumed a major role in ongoing efforts to eliminate onchocerciasis. Using either a single *O. volvulus* recombinant protein or a pool of different recombinant proteins, specific subgroups in a population, particularly children, can be screened for the presence of antibodies specific to these proteins, with an absence of antibodies indicating the interruption of transmission [[10-18](#_ENREF_10)]. The possibility of cross-reactivity to other antigens, particularly other filarial parasites is potentially problematic in some epidemiological settings.

Mapping *L. loa* prevalence, in the context of possible ivermectin-induced SAEs, based on antibody response to a recombinant protein has been described [[19](#_ENREF_19)]. When antibody response to this recombinant protein was combined with a luciferase immunoprecipitation system (LIPS), a significant improvement in sensitivity and specificity was observed [[16](#_ENREF_16)].

For LF, sero-responsiveness to *B. malayi* adult worm antigens has been widely used in clinical diagnostic settings. When the antibody response to whole parasite antigen is measured, cross-reactivity with other nematode infections may reduce assay specificity, and sero-reversion may be delayed following cure. Assays where an antibody response of the IgG4 subclass is measured results in increased specificity compared to measurement of total IgG responses [[20](#_ENREF_20)], and assays measuring this subclass response have shown utility in control programmes [[11](#_ENREF_11)]. As no circulating antigen test is available for brugian filariasis (see below), a dipstick antibody test for diagnosis of *B. malayi* infection has been developed, based on antibody response to a recombinant *B. malayi* protein BmR1 [[21](#_ENREF_21),[22](#_ENREF_22)]. This rapid test shows promise as a tool in the elimination program for *B. malayi.*

***Antigen Detection***

A major advance in diagnostic capacity for bancroftian filariasis was the development of antigen capture assays that enable detection of circulating antigen of *W. bancrofti* [[23](#_ENREF_23),[24](#_ENREF_24)]. Such tests have enabled case-based diagnosis without the need for nocturnal blood collection, assessment of cure through clearance of antigenaemia [[25](#_ENREF_25)], and epidemiological mapping of endemicity [[26](#_ENREF_26),[27](#_ENREF_27)]. These tests are showing good utility as tools for elimination efforts [[28](#_ENREF_28)]. Despite some effort, no equivalent tests have been developed for diagnosis of brugian filariasis, loiasis or onchocerciasis.

A recent significant advance has occurred through the use of modern proteomic technology to identify biomarkers in the blood of infected individuals. When this approach was recently undertaken using 73 sera and plasma samples from patients with onchocerciasis, fourteen candidate biomarkers were identified [[29](#_ENREF_29)]. It is of interest that only four of these fourteen were proteins, the traditional target of antibody based diagnostic assays. This indicates both an opportunity in terms of untapped biomarkers, but also a challenge, as traditional diagnostic tests are not configured to detect lipids, sugars or prostanoid molecules.

***Molecular Diagnosis***

PCR-based tests have assumed a major place in the diagnostic arsenal for filariases, particularly for assessment of infection in mosquito or fly vectors (see below). PCR-based diagnosis of skin scrapings for *O. volvulus* [[30](#_ENREF_30)] is more sensitive than parasitological examination of skin snips, as is PCR of blood for brugian and bancroftian filariae [[31](#_ENREF_31),[32](#_ENREF_32)].

***Vectors***

Molecular xenomonitoring by PCR testing of fly or mosquito vector population samples has become an invaluable tool for epidemiological monitoring in onchocerciasis [[33](#_ENREF_33)], and filariasis including in elimination programmes [[28](#_ENREF_28)]. However, as the intensity of infection decreases, the number of insects that would be required to be analyzed to accurately quantify transmission intensity increases concomitantly [[34](#_ENREF_34)]. In practice however, the number of vectors that elimination programmes recommend to be analyzed remains relatively constant, with specific sampling targets being proposed in elimination settings. It is necessary to account for a number of important technical considerations if xenomonitoring is used to monitor transmission. For example, as only parous vectors would have the potential to transmit infection (in contrast to nulliparous vectors caught at their first feeding), the proportion of parous females should be a consideration in any analysis of vector infection. The use of xenomonitoring, in conjunction with molecular biological methods, has been developed to determine whether savannah or forest strains of *O. volvulus* are being transmitted as these strain differences have implications for onchocercal blinding and skin disease. Molecular test can also be undertaken to determine whether *Onchocerca* larval stages in the blackfly vector are *O. volvulus* or other *Onchocerca* species that only infect non-human animals but are transmitted by the same vectors (e.g. *O. ochengi*).

Another application of xenodiagnostic tests has been the use of clean insects to feed on people for detection of submicroscopic infections. Such testing may be more sensitive than standard parasitological diagnosis for incompletely understood reasons including the hypothesis that mf may be attracted to components of some vectors’ saliva, and the larger amount of sampling achieved using such methods [[35](#_ENREF_35),[36](#_ENREF_36)]. Because of ethical considerations, traps for vectors may represent an alternative to using human bait. For some species of mosquito vectors of LF, indoor resting collection and CDC light traps may be efficient means of collecting vectors, in particular when the numbers of mosquitoes caught in light traps correlates positively and significantly with the numbers that would be attracted by a person sleeping under a (non-insecticide) impregnated net [[37](#_ENREF_37)]. Monkswood light traps or Béllec sticky traps, positioned close to *Simulium* breeding sites, can effectively trap gravid blackflies as they seek to oviposit [[38-40](#_ENREF_38)]. However, they do not help estimate biting (landing) rates, necessary for calculation of annual infective biting rates and transmission potentials.

**References**

1. Collins RC, Brandling-Bennett AD, Holliman RB, Campbell CC, Darsie RF (1980) Parasitological diagnosis of onchocerciasis: comparisons of incubation media and incubation times for skin snips. Am J Trop Med Hyg 29: 35-41.

2. Taylor HR, Keyvan-Larijani E, Newland HS, White AT, Greene BM (1987) Sensitivity of skin snips in the diagnosis of onchocerciasis. Trop Med Parasitol 38: 145-147.

3. Stingl P, Ross M, Gibson DW, Ribas J, Connor DH (1984) A diagnostic "patch test" for onchocerciasis using topical diethylcarbamazine.
Trans R Soc Trop Med Hyg 78: 254-258.

4. Ozoh G, Boussinesq M, Bissek AC, Kobangue L, Kombila M, et al. (2007) Evaluation of the diethylcarbamazine patch to evaluate onchocerciasis endemicity in Central Africa. Trop Med Int Health 12: 123-129.

5. McMahon JE (1982) The examination--time/dose interval in the provocation of nocturnally periodic microfilariae of Wuchereria bancrofti with diethylcarbamazine and the practical uses of the test. Tropenmed Parasitol 33: 28-30.

6. Duerr HP, Raddatz G, Eichner M (2008) Diagnostic value of nodule palpation in onchocerciasis. Trans R Soc Trop Med Hyg 102: 148-154.

7. Katabarwa M, Lakwo T, Habumogisha P, Richards F, Eberhard M (2008) Could neurocysticercosis be the cause of "onchocerciasis-associated" epileptic seizures?
Am J Trop Med Hyg 78: 400-401.

8. Mand S, Marfo-Debrekyei Y, Debrah A, Buettner M, Batsa L, et al. (2005) Frequent detection of worm movements in onchocercal nodules by ultrasonography.
Filaria J 4: 1.

9. Palumbo E (2008) Filariasis: diagnosis, treatment and prevention.
Acta Biomed 79: 106-109.

10. Gonzalez RJ, Cruz-Ortiz N, Rizzo N, Richards J, Zea-Flores G, et al. (2009) Successful interruption of transmission of Onchocerca volvulus in the Escuintla-Guatemala focus, Guatemala. PLoS Negl Trop Dis 3: e404.

11. Lindblade KA, Arana B, Zea-Flores G, Rizzo N, Porter CH, et al. (2007) Elimination of Onchocercia volvulus transmission in the Santa Rosa focus of Guatemala.
Am J Trop Med Hyg 77: 334-341.

12. Rodriguez-Perez MA, Dominguez-Vazquez A, Mendez-Galvan J, Sifuentes-Rincon AM, Larralde-Corona P, et al. (2003) Antibody detection tests for *Onchocerca volvulus*: comparison of the sensitivity of a cocktail of recombinant antigens used in the indirect enzyme-linked immunosorbent assay with a rapid-format antibody card test. Trans R Soc Trop Med Hyg 97: 539-541.

13. Weil GJ, Steel C, Liftis F, Li BW, Mearns G, et al. (2000) A rapid-format antibody card test for diagnosis of onchocerciasis. J Infect Dis 182: 1796-1799.

14. Rodriguez-Perez MA, Lizarazo-Ortega C, Hassan HK, Dominguez-Vasquez A, Mendez-Galvan J, et al. (2008) Evidence for suppression of *Onchocerca volvulus* transmission in the Oaxaca focus in Mexico. Am J Trop Med Hyg 78: 147-152.

15. Rodriguez-Perez MA, Lutzow-Steiner MA, Segura-Cabrera A, Lizarazo-Ortega C, Dominguez-Vazquez A, et al. (2008) Rapid suppression of *Onchocerca volvulus* transmission in two communities of the Southern Chiapas focus, Mexico, achieved by quarterly treatments with Mectizan. Am J Trop Med Hyg 79: 239-244.

16. Burbelo PD, Ramanathan R, Klion AD, Iadarola MJ, Nutman TB (2008) Rapid, novel, specific, high-throughput assay for diagnosis of *Loa loa* infection. J Clin Microbiol 46: 2298-2304.

17. Burbelo PD, Leahy HP, Iadarola MJ, Nutman TB (2009) A four-antigen mixture for rapid assessment of *Onchocerca volvulus* infection. PLoS Negl Trop Dis 3: e438.

18. Lipner EM, Dembele N, Souleymane S, Alley WS, Prevots DR, et al. (2006) Field applicability of a rapid-format anti-Ov-16 antibody test for the assessment of onchocerciasis control measures in regions of endemicity. J Infect Dis 194: 216-221.

19. Klion AD, Vijaykumar A, Oei T, Martin B, Nutman TB (2003) Serum immunoglobulin G4 antibodies to the recombinant antigen, Ll-SXP-1, are highly specific for *Loa loa* infection. J Infect Dis 187: 128-133.

20. Lal RB, Ottesen EA (1988) Enhanced diagnostic specificity in human filariasis by IgG4 antibody assessment. J Infect Dis 158: 1034-1037.

21. Rahmah N, Lim BH, Khairul Anuar A, Shenoy RK, Kumaraswami V, et al. (2001) A recombinant antigen-based IgG4 ELISA for the specific and sensitive detection of *Brugia malayi* infection. Trans R Soc Trop Med Hyg 95: 280-284.

22. Rahmah N, Shenoy RK, Nutman TB, Weiss N, Gilmour K, et al. (2003) Multicentre laboratory evaluation of Brugia Rapid dipstick test for detection of brugian filariasis. Trop Med Int Health 8: 895-900.

23. More SJ, Copeman DB (1990) A highly specific and sensitive monoclonal antibody-based ELISA for the detection of circulating antigen in bancroftian filariasis.
Trop Med Parasitol 41: 403-406.

24. Weil GJ, Jain DC, Santhanam S, Malhotra A, Kumar H, et al. (1987) A monoclonal antibody-based enzyme immunoassay for detecting parasite antigenemia in bancroftian filariasis. J Infect Dis 156: 350-355.

25. McCarthy JS, Guinea A, Weil GJ, Ottesen EA (1995) Clearance of circulating filarial antigen as a measure of the macrofilaricidal activity of diethylcarbamazine in *Wuchereria bancrofti* infection. J Infect Dis 172: 521-526.

26. Gyapong JO, Kyelem D, Kleinschmidt I, Agbo K, Ahouandogbo F, et al. (2002) The use of spatial analysis in mapping the distribution of bancroftian filariasis in four West African countries. Ann Trop Med Parasitol 96: 695-705.

27. Onapa AW, Simonsen PE, Baehr I, Pedersen EM (2005) Rapid assessment of the geographical distribution of lymphatic filariasis in Uganda, by screening of schoolchildren for circulating filarial antigens. Ann Trop Med Parasitol 99: 141-153.

28. Ramzy RM, El Setouhy M, Helmy H, Ahmed ES, Abd Elaziz KM, et al. (2006) Effect of yearly mass drug administration with diethylcarbamazine and albendazole on bancroftian filariasis in Egypt: a comprehensive assessment. Lancet 367: 992-999.

29. Denery JR, Nunes AA, Hixon MS, Dickerson TJ, Janda KD (2010) Metabolomics-based discovery of diagnostic biomarkers for onchocerciasis. PLoS Negl Trop Dis 4.

30. Toe L, Boatin BA, Adjami A, Back C, Merriweather A, et al. (1998) Detection of *Onchocerca volvulus* infection by O-150 polymerase chain reaction analysis of skin scratches. J Infect Dis 178: 282-285.

31. Zhong M, McCarthy J, Bierwert L, Lizotte-Waniewski M, Chanteau S, et al. (1996) A polymerase chain reaction assay for detection of the parasite *Wuchereria bancrofti* in human blood samples. Am J Trop Med Hyg 54: 357-363.

32. Fink DL, Fahle GA, Fischer S, Fedorko DF, Nutman TB (2011) Toward molecular parasitologic diagnosis: enhanced diagnostic sensitivity for filarial infections in mobile populations. J Clin Microbiol 49: 42-47.

33. Boatin BA, Richards FO, Jr. (2006) Control of onchocerciasis.
Adv Parasitol 61: 349-394.

34. Basáñez MG, Rodriguez-Perez MA, Reyes-Villanueva F, Collins RC, Rodriguez MH (1998) Determination of sample sizes for the estimation of *Onchocerca volvulus* (Filarioidea: Onchocercidae) infection rates in biting populations of Simulium ochraceum s.l. (Diptera: Simuliidae) and its application to ivermectin control programs. J Med Entomol 35: 745-757.

35. Davies JB, Lujan R, Lopez-Martinez LA, Paniagua-Alvarez AJ, Morales-Hernandez BE, et al. (1997) Assessment of vector microfilarial uptake as a comparatively non-invasive technique for monitoring onchocerciasis treatment campaigns in the Americas. Trop Med Int Health 2: 348-355.

36. Shelley AJ, Pinger RR, Moraes MA, Hayes J (1979) Concentration of microfilariae of *Onchocerca volvulus* by *Simulium sanguineum* during feeding: use in mapping parasite distribution in the skin. J Med Entomol 16: 48-51.

37. Magbity EB, Magbity EB, Lines JD, Marbiah MT, David K, et al. (2002) How reliable are light traps in estimating biting rates of adult *Anopheles gambiae* s.l. (Diptera: Culicidae) in the presence of treated bed nets? Bull Entomol Res 92: 71-76.

38. Service MW (1979) Light trap collections of ovipositing *Simulium squamosum* in Ghana. Ann Trop Med Parasitol 73: 487-490.

39. Walsh JF (1978) Light trap studies on *Simulium damnosum* s.l. in Northern Ghana. Tropenmed Parasitol 29: 492-496.

40. Walsh JF (1980) Sticky trap studies on *Simulium damnosum* s.l. in northern Ghana. Tropenmed Parasitol 31: 479-486.
