# Supplementary material for: A Research Agenda for Helminth Diseases of Humans: Diagnostics for Control and Elimination Programmes
Source: PLoS Negl Trop Dis. 2012 Apr 24;6(4):e1601. doi: 10.1371/journal.pntd.0001601 (PMC3335877; doi:10.1371/journal.pntd.0001601)
Supplement: Text S3 — Diagnostics for Schistosomiasis. (DOCX) [file pntd.0001601.s003.docx]

**Text S3. Diagnostics for Schistosomiasis**

***Parasitological Diagnosis***

Parasitological diagnosis of *Schistosoma haematobium* infection is readily undertaken by urine filtration. While urine is easily collected, due to the circadian pattern of egg excretion, specimens should ideally be collected between 10 am and 2 pm, and preferably after physical exercise [[1](#_ENREF_1)]. Diagnosis of intestinal schistosomiasis (*S. mansoni* and *S. japonicum*) is generally made by examination of stool specimens. The Kato-Katz thick smear method is the standard method recommended by the World Health Organization (WHO) for both qualitative and quantitative diagnosis of intestinal schistosomiasis. Its main advantage is that it is highly specific, relatively simple and inexpensive, even under field conditions. Furthermore, it produces semi-quantitative egg counts that can be used as surrogates of infection intensity. However, like many other parasitological tests, if only a single Kato-Katz slide is prepared from a single stool specimen, sensitivity is reduced, particularly in light infections. This leads to a marked underestimation of the prevalence of infection especially in low prevalence areas [[2](#_ENREF_2),[3](#_ENREF_3)], where infection intensity is low [[4](#_ENREF_4)], and can confound confirmation of cure assessment after chemotherapy [[5-8](#_ENREF_5)]. As for many other helminth infections, the use of parasitological diagnosis as the ‘gold standard’ makes other diagnostic test results (incorrectly) appear as false [[9](#_ENREF_9)]. To overcome the lack of sensitivity of the parasitological methods (urine filtration and Kato-Katz technique) in situations of low worm burden, replicate urine or stool samples, or a number of Kato-Katz slides prepared from a single (or sequential) stool sample, are required. However, this increases costs, may hamper the survey for the need of repeated samples, and would complicate control strategies based on screen and treat [[10](#_ENREF_10)]. In China, where the aim of some control programmes is the elimination of *S. japonicum*, a two-step diagnostic approach has been implemented, entailing initial serodiagnostic screening (see below), followed by stool examination using the Kato-Katz technique in seropositive individuals [[11](#_ENREF_11)]. A number of specific problems pertain to the parasitological diagnosis of *S. japonicum.* These include the nondescript round shape of the eggs that can be confused with protozoan cysts, air bubbles, pollen etc.

For *S. haematobium* infection, the presence of micro- or macrohaematuria has enabled the development and validation of a range of indirect diagnostic tests useful for epidemiological mapping of prevalence, such as dipstick methods which detect micro- and macro-haematuria. Simple interview methods to ascertain a history of haematuria have also been shown to be useful. For example, the WHO-supported Red Urine Study and others have established the utility of a simple oral questionnaire for history of haematuria, to estimate the prevalence of infection among school-age children [[12-15](#_ENREF_12)].

***Antibody Tests***

Patent schistosome infection is highly immunogenic, and anti-schistosome antibodies can be readily detected using a wide range of immunodiagnostic techniques. Currently, the ELISA technique, using soluble egg antigen (SEA) as the target, is the most widely used technique [[9](#_ENREF_9),[16-18](#_ENREF_16)]. Other techniques, such as Dipstick Dye Immunoassays (DDIA) are also used [[11](#_ENREF_11)]. However, serodiagnosis of schistosomiasis suffers from a number of drawbacks common to antibody detection techniques for parasite infection [[9](#_ENREF_9),[10](#_ENREF_10)]. These include the difficulty distinguishing active from past infection, with parasite-specific antibodies remaining long time after cure, and inability to measure infection intensity. Of interest, in an experimental study of *S. japonicum* in a pig model, parasite-specific IgG levels decreased below the diagnostic threshold 12 weeks after praziquantel treatment. [[19](#_ENREF_19)].

Nevertheless, immunodiagnostic techniques remain the best available methods for diagnosis in areas of low intensity of infection where the sensitivity and specificity of these methods appear to be satisfactory [[20](#_ENREF_20)]. A range of approaches to improving the accuracy of immunodiagnostic assays have been described. These include using specific parasite extracts such as cercarial extract [[21](#_ENREF_21)], using recombinant proteins as immunodiagnostic targets [[22](#_ENREF_22)], or using a pool of synthetic peptides selected on the basis of the amino acid sequence of proteins from different antigenic preparations of *S. mansoni* [[23](#_ENREF_23)]. An alternative approach that has been used with success in China is to combine information from serological surveys with parasitological data, and to use a Bayesian statistical approach to develop accurate epidemiological maps of infection prevalence [[24](#_ENREF_24)].

***Antigen Detection***

Schistosome antigens are present in serum and urine of infected subjects [[25](#_ENREF_25)]. According to their migratory behaviour in immunoelectrophoresis they are commonly referred to as circulating anodic antigens (CAA) and circulating cathodic antigens (CCA). These two circulating adult worm antigens are the basis of antigen capture immunoassays [[26](#_ENREF_26)]. Measurement of CAA in the blood, serum, and urine by ELISA-based assays is sensitive, specific and much less variable than egg counts [[27](#_ENREF_27)]. The CCA assay has been further developed as a point of care (POC) urine ELISA dipstick [[28](#_ENREF_28)]. In field evaluation studies, the test was unexpectedly insensitive for detection of *S. haematobium* infections [[29-31](#_ENREF_29)], but performed relatively well for *S. mansoni* infection [[30](#_ENREF_30),[32](#_ENREF_32)]. There was a correlation between intensity of *S. mansoni* infection, as measured by egg counts and CCA concentration [[30](#_ENREF_30)]. Another hurdle limiting further use of the CCA test, even within an area monoendemic for intestinal schistosomiasis is the cost of the dipsticks, currently more than US$2 per test [[31](#_ENREF_31)].

***Molecular Diagnosis***

The application of PCR as a technique for the detection of schistosomiasis has been explored for *S. mansoni* and *S. japonicum* in human faeces [[33](#_ENREF_33),[34](#_ENREF_34)] and urine [[35](#_ENREF_35)]. The technique has been evaluated in areas of medium and low intensity of infection. While this methodology has proved to be highly sensitive and specific, its application is currently limited, as it requires significant infrastructure, financial resources, and skilled personnel [[4](#_ENREF_4),[36](#_ENREF_36),[37](#_ENREF_37)].

***Snail Intermediate Hosts***

The extent of geographical distribution of schistosomiasis is limited by the distribution of susceptible snail intermediate hosts. Susceptibility to human schistosome infection varies among snails of a given species, with incompletely understood factors such as snail age and intraspecies genetic variation influencing susceptibility [[38](#_ENREF_38)]. PCR-based methodologies have been developed for a range of purposes in snails, including assisting their morphological classification; determining their infection status; detecting infection in the pre-patent period; identifying which species of trematode the snail is infected with; and detecting infection in dead snails [[39](#_ENREF_39)].

**References**

1. Feldmeier H (1993) Diagnosis in Human schistosomiasis. In: Jordan P, Webbe G, Sturrock R, editors. Human Schistosomiasis. Oxon: CAB International. pp. 271-303.

2. Lin DD, Liu JX, Liu YM, Hu F, Zhang YY, et al. (2008) Routine Kato-Katz technique underestimates the prevalence of *Schistosoma japonicum*: a case study in an endemic area of the People's Republic of China. Parasitol Int 57: 281-286.

3. Yu JM, de Vlas SJ, Jiang QW, Gryseels B (2007) Comparison of the Kato-Katz technique, hatching test and indirect hemagglutination assay (IHA) for the diagnosis of *Schistosoma japonicum* infection in China. Parasitology international 56: 45-49.

4. Lier T, Johansen MV, Hjelmevoll SO, Vennervald BJ, Simonsen GS (2008) Real-time PCR for detection of low intensity *Schistosoma japonicum* infections in a pig model. Acta Trop 105: 74-80.

5. Berhe N, Medhin G, Erko B, Smith T, Gedamu S, et al. (2004) Variations in helminth faecal egg counts in Kato-Katz thick smears and their implications in assessing infection status with *Schistosoma mansoni*. Acta Trop 92: 205-212.

6. Booth M, Vounatsou P, N'Goran E K, Tanner M, Utzinger J (2003) The influence of sampling effort and the performance of the Kato-Katz technique in diagnosing *Schistosoma mansoni* and hookworm co-infections in rural Cote d'Ivoire. Parasitology 127: 525-531.

7. Kongs A, Marks G, Verle P, Van der Stuyft P (2001) The unreliability of the Kato-Katz technique limits its usefulness for evaluating *S. mansoni* infections.
Trop Med Int Health 6: 163-169.

8. Utzinger J, Booth M, N'Goran EK, Muller I, Tanner M, et al. (2001) Relative contribution of day-to-day and intra-specimen variation in faecal egg counts of *Schistosoma mansoni* before and after treatment with praziquantel.
Parasitology 122: 537-544.

9. Doenhoff MJ, Chiodini PL, Hamilton JV (2004) Specific and sensitive diagnosis of schistosome infection: can it be done with antibodies? Trends Parasitol 20: 35-39.

10. Rabello A, Enk M (2006) Progress towards the detection of schistosomiasis. In: WHO, editor. Report of the scientific working group meeting on schistosomiasis for the special programme for research and training in tropical diseases. Geneva: WHO. pp. 67-71.

11. Zhu YC (2005) Immunodiagnosis and its role in schistosomiasis control in China: a review. Acta Trop 96: 130-136.

12. Guyatt H, Brooker S, Lwambo NJ, Siza JE, Bundy DA (1999) The performance of school-based questionnaires of reported blood in urine in diagnosing *Schistosoma haematobium* infection: patterns by age and sex. Trop Med Int Health 4: 751-757.

13. Lengeler C, de Savigny D, Mshinda H, Mayombana C, Tayari S, et al. (1991) Community-based questionnaires and health statistics as tools for the cost-efficient identification of communities at risk of urinary schistosomiasis.
Int J Epidemiol 20: 796-807.

14. Lengeler C, Utzinger J, Tanner M (2002) Questionnaires for rapid screening of schistosomiasis in sub-Saharan Africa. Bull World Health Organ 80: 235-242.

15. WHO (1998) Report of the WHO informal consultation on schistosomiasis control. Geneva.

16. McLaren M, Draper CC, Roberts JM, Minter-Goedbloed E, Ligthart GS, et al. (1978) Studies on the enzyme linked immunosorbent assay (ELISA) test for *Schistosoma mansoni* infections. Ann Trop Med Parasitol 72: 243-253.

17. Mott KE, Dixon H (1982) Collaborative study on antigens for immunodiagnosis of schistosomiasis. Bull World Health Organ 60: 729-753.

18. Mott KE, Dixon H, Carter CE, Garcia E, Ishii A, et al. (1987) Collaborative study on antigens for immunodiagnosis of *Schistosoma japonicum* infection.
Bull World Health Organ 65: 233-244.

19. Johansen MV, Bogh HO, Nansen P, Christensen NO (2000) Schistosoma japonicum infection in the pig as a model for human schistosomiasis japonica.
Acta Trop 76: 85-99.

20. Goncalves MM, Barreto MG, Peralta RH, Gargioni C, Goncalves T, et al. (2006) Immunoassays as an auxiliary tool for the serodiagnosis of *Schistosoma mansoni* infection in individuals with low intensity of egg elimination. Acta Trop 100: 24-30.

21. Chand MA, Chiodini PL, Doenhoff MJ (2010) Development of a new assay for the diagnosis of schistosomiasis, using cercarial antigens.
Trans R Soc Trop Med Hyg 104: 255-258.

22. Zhou XH, Wu JY, Huang XQ, Kunnon SP, Zhu XQ, et al. (2010) Identification and characterization of *Schistosoma japonicum* Sjp40, a potential antigen candidate for the early diagnosis of schistosomiasis. Diagn Microbiol Infect Dis 67: 337-345.

23. de Oliveira EJ, Kanamura HY, Takei K, Hirata RD, Valli LC, et al. (2008) Synthetic peptides as an antigenic base in an ELISA for laboratory diagnosis of schistosomiasis mansoni. Trans R Soc Trop Med Hyg 102: 360-366.

24. Wang XH, Wu XH, Zhou XN (2006) Bayesian estimation of community prevalences of *Schistosoma japonicum* infection in China. Int J Parasitol 36: 895-902.

25. Deelder AM, Klappe HT, van den Aardweg GJ, van Meerbeke EH (1976) *Schistosoma mansoni*: demonstration of two circulating antigens in infected hamsters. Exp Parasitol 40: 189-197.

26. Al-Sherbiny MM, Osman AM, Hancock K, Deelder AM, Tsang VC (1999) Application of immunodiagnostic assays: detection of antibodies and circulating antigens in human schistosomiasis and correlation with clinical findings.
Am J Trop Med Hyg 60: 960-966.

27. Vennervald BJ, Ouma JH, Butterworth AE (1998) Morbidity in schistosomiasis: assessment, mechanisms and control. Parasitol Today 14: 385-390.

28. van Dam GJ, Wichers JH, Ferreira TM, Ghati D, van Amerongen A, et al. (2004) Diagnosis of schistosomiasis by reagent strip test for detection of circulating cathodic antigen. J Clin Microbiol 42: 5458-5461.

29. Ayele B, Erko B, Legesse M, Hailu A, Medhin G (2008) Evaluation of circulating cathodic antigen (CCA) strip for diagnosis of urinary schistosomiasis in Hassoba school children, Afar, Ethiopia. Parasite 15: 69-75.

30. Stothard JR, Kabatereine NB, Tukahebwa EM, Kazibwe F, Rollinson D, et al. (2006) Use of circulating cathodic antigen (CCA) dipsticks for detection of intestinal and urinary schistosomiasis. Acta Trop 97: 219-228.

31. Stothard JR, Sousa-Figueiredo JC, Standley C, Van Dam GJ, Knopp S, et al. (2009) An evaluation of urine-CCA strip test and fingerprick blood SEA-ELISA for detection of urinary schistosomiasis in schoolchildren in Zanzibar. Acta Trop 111: 64-70.

32. Legesse M, Erko B (2007) Field-based evaluation of a reagent strip test for diagnosis of *Schistosoma mansoni* by detecting circulating cathodic antigen in urine before and after chemotherapy. Trans R Soc Trop Med Hyg 101: 668-673.

33. Lier T, Simonsen GS, Wang T, Lu D, Haukland HH, et al. (2009) Real-time polymerase chain reaction for detection of low-intensity *Schistosoma japonicum* infections in China. Am J Trop Med Hyg 81: 428-432.

34. ten Hove RJ, Verweij JJ, Vereecken K, Polman K, Dieye L, et al. (2008) Multiplex real-time PCR for the detection and quantification of *Schistosoma mansoni* and *S. haematobium* infection in stool samples collected in northern Senegal.

Trans R Soc Trop Med Hyg 102: 179-185.

35. Obeng BB, Aryeetey YA, de Dood CJ, Amoah AS, Larbi IA, et al. (2008) Application of a circulating-cathodic-antigen (CCA) strip test and real-time PCR, in comparison with microscopy, for the detection of *Schistosoma haematobium* in urine samples from Ghana. Ann Trop Med Parasitol 102: 625-633.

36. Lier T, Simonsen GS, Haaheim H, Hjelmevoll SO, Vennervald BJ, et al. (2006) Novel real-time PCR for detection of *Schistosoma japonicum* in stool. Southeast Asian J Trop Med Public Health 37: 257-264.

37. Pontes LA, Oliveira MC, Katz N, Dias-Neto E, Rabello A (2003) Comparison of a polymerase chain reaction and the Kato-Katz technique for diagnosing infection with *Schistosoma mansoni*. Am J Trop Med Hyg 68: 652-656.

38. Richards CS, Shade PC (1987) The genetic variation of compatibility in *Biomphalaria glabrata* and *Schistosoma mansoni*. J Parasitol 73: 1146-1151.

39. Caldeira RL, Jannotti-Passos LK, Carvalho OS (2009) Molecular epidemiology of Brazilian Biomphalaria: a review of the identification of species and the detection of infected snails. Acta Trop 111: 1-6.
