# Supplementary material for: A Research Agenda for Helminth Diseases of Humans: Diagnostics for Control and Elimination Programmes
Source: PLoS Negl Trop Dis. 2012 Apr 24;6(4):e1601. doi: 10.1371/journal.pntd.0001601 (PMC3335877; doi:10.1371/journal.pntd.0001601)
Supplement: Text S4 — Diagnostics for Cystic Hydatid Disease. (DOCX) [file pntd.0001601.s004.docx]

**Text S4. Diagnostics for cystic hydatid diseases**

***Parasitological Diagnosis***

Capturing dogs to collect stool samples for parasitological stool examination is a highly inefficient strategy for monitoring infection prevalence since the dog population is dispersed and of course not compliant. Although dog stools can be collected from the environment and examined for taeniid eggs, making a species-specific diagnosis is difficult because taeniid eggs from several species including *Taenia hydatigena* and *Taenia multiceps* are morphologically indistinguishable from those of *Echinococcus granulosus.* In individual settings it is possible to undertake a purge with arecoline to examine post-purge stools for *E. granulosus* worms, and confirm the diagnosis and parasite burden. This complicated, resource-intensive procedure has the additional drawbacks of discomfort for the animals and biosafety risks for humans if not properly performed [[1](#_ENREF_1)].

***Antibody Tests***

No serological tests are available for diagnosis of *E. granulosus* infection of dogs with the adult tapeworm. Although many antibody assays exist for diagnosis of CHD in humans and other intermediate hosts, their performance is suboptimal and their role is restricted to cross-sectional assessment of the level of infection in a given herd or population, or to support clinical or imaging-based diagnosis in specific cases [[2](#_ENREF_2)].

***Antigen Detection***

While serum antigen detection is of minimal or no use in canine echinococcosis or CHD, stool antigen detection is a useful tool to assess infection levels in dog populations [[1](#_ENREF_1)], and antigen capture assays using polyclonal antibodies have been widely used in control programmes. An innovative approach that was used used in Rio Negro, Argentina entailed collection of dog stool samples from the environment followed by examined by ELISA coproantigen detection and copro-western blot to provide epidemiological information on the level of infection in individual farms [[1](#_ENREF_1)].

***Molecular Diagnosis***

Diagnostic stool PCR methods have been developed and are in use in control programmes and research settings [[3](#_ENREF_3)]. The farm-based approach described above may prove more pragmatic than individual dog sampling or diagnosis.

**References**

1. Guarnera EA, Santillan G, Botinelli R, Franco A (2000) Canine echinococcosis: an alternative for surveillance epidemiology. Vet Parasitol 88: 131-134.

2. Brunetti E, Garcia HH, Junghanss T, on behalf of the members of the international CE workshop in Lima / Peru 2009 (2011 (in press)) Cystic echinococcosis: chronic, complex, and still neglected. PLoS Negl Trop Dis.

3. Boufana BS, Campos-Ponce M, Naidich A, Buishi I, Lahmar S, et al. (2008) Evaluation of three PCR assays for the identification of the sheep strain (genotype 1) of *Echinococcus granulosus* in canid feces and parasite tissues.
Am J Trop Med Hyg 78: 777-783.
