# Supplementary material for: A Research Agenda for Helminth Diseases of Humans: Diagnostics for Control and Elimination Programmes
Source: PLoS Negl Trop Dis. 2012 Apr 24;6(4):e1601. doi: 10.1371/journal.pntd.0001601 (PMC3335877; doi:10.1371/journal.pntd.0001601)
Supplement: Text S5 — Diagnostics for Taeniasis Cysticercosis. (DOCX) [file pntd.0001601.s005.docx]

**Text S5. Diagnosis of taeniasis and cysticercosis**

***Parasitological Diagnosis***

Stool microscopy for diagnosis of taeniasis is inefficient and thus it is not recommended unless there is a specific indication and no suitable alternative. Even with multiple samples and concentration of large volumes of stool sample, sensitivity of stool microscopy does not exceed 60 to 70% [[1](#_ENREF_1)]. As the eggs of *T. solium* are morphologically identical to those of *T. saginata*, the finding of *Taenia* eggs should be reported as *Taenia spp*. eggs. While it is theoretically possible to distinguish proglottids of *T. saginata* from those of *T. solium* by counting the number of uterine branches, this is not an easy test to perform and proglottid material is infrequently available. However, given that the prevalence of infection with either species is usual low, the role of parasitologic diagnosis in control programmes is relatively minor. For diagnosis of cysticercosis, histological confirmation of excised cysts is rarely required, nor easily undertaken except in a small proportion of patients with subcutaneous nodules where biopsy can provide diagnostic support.

***Antibody Tests***

A Western blot assay using purified parasite glycoprotein antigens is the assay of choice for cysticercosis [[2](#_ENREF_2)]. Diagnosing cysticercosis-infected humans or pigs, however, does not contribute to control of transmission except for detecting humans who may simultaneously carry a tapeworm. Antibody assays in pigs are discussed below. If available, the most important use of a serodiagnostic assay for control would be an assay specific for diagnosis of humans carrying an adult tapeworm (and who are thus a source of transmission). Although such a test has been described [[3](#_ENREF_3)], there is only insufficient information on the longevity of antibodies. If an antibody response persists long after the death of the tapeworm, uninfected individuals with residual antibodies may far outnumber current tapeworm carriers, affecting test performance in programmatic settings. Of note however, both of the above described tests require parasitic cysts or tapeworm excretory/secretory material as a source of antigen. Assays using recombinant or synthetic antigens if availablewould be more suitable.

***Antigen Detection***

While a monoclonal antibody-based ELISA assays to detect parasite antigen circulating in the blood have been recently re-visited [[4-6](#_ENREF_4)], and represent useful tools for clinical follow up of patients with neurocysticercosis (NCC), such tests are not of great assistance in control programmes. Stool antigen detection, however, may play a major role. Such tests are appropriate for screening of large numbers of stool samples, and are more sensitive than parasitological stool examination [[7](#_ENREF_7)]. As such ELISA-based coproantigen detection is to date the best diagnostic tool for human taeniasis. However, this test cannot distinguish *T. solium* from *T. saginata* infection, and its availability beyond research settings is very limited.

***Molecular Diagnosis***

Stool PCR and loop-mediated isothermal amplification (LAMP) can distinguish between *T. saginata* and *T. solium*. However, these assays are not routinely available, and their usefulness for case detection in field conditions has yet to be established [[8-10](#_ENREF_8)].

***Intermediate Hosts***

Diagnosis in pigs (porcine cysticercosis) can be made by tongue inspection, antibody or antigen detection, or by postmortem inspection at slaughterhouses. Rapid lingual examination for cysts is an inexpensive but insensitive test [[11](#_ENREF_11)]. Likewise, diagnosis by detection of cysts at slaughter of pigs is also insensitive. Techniques developed for the immunodiagnosis of cysticercosis in humans have been adapted for diagnosis of *T. solium* infection of pigs, including both antibody [[12-14](#_ENREF_12)] and antigen detection. These tests are more sensitive than lingual examination for use in prevalence surveys and control programmes [[11](#_ENREF_11)]. While many pigs are seropositive but have no cysts at necropsy (most likely due to exposure antibodies or aborted infections), strong antibody reactions in pigs are generally associated with active cysticercosis. However, the sensitivity of these immunodiagnostic techniques is reduced in pigs with low infection intensity [[15](#_ENREF_15)].

**References**

1. Allan JC, Mencos F, Garcia-Noval J, Sarti E, Flisser A, et al. (1993) Dipstick dot ELISA for the detection of Taenia coproantigens in humans.
Parasitology 107 ( Pt 1): 79-85.

2. Tsang VC, Brand JA, Boyer AE (1989) An enzyme-linked immunoelectrotransfer blot assay and glycoprotein antigens for diagnosing human cysticercosis (*Taenia solium*).
J Infect Dis 159: 50-59.

3. Wilkins PP, Allan JC, Verastegui M, Acosta M, Eason AG, et al. (1999) Development of a serologic assay to detect *Taenia solium* taeniasis.
Am J Trop Med Hyg 60: 199-204.

4. Brandt JR, Geerts S, De Deken R, Kumar V, Ceulemans F, et al. (1992) A monoclonal antibody-based ELISA for the detection of circulating excretory-secretory antigens in *Taenia saginata* cysticercosis. Int J Parasitol 22: 471-477.

5. Garcia HH, Parkhouse RM, Gilman RH, Montenegro T, Bernal T, et al. (2000) Serum antigen detection in the diagnosis, treatment, and follow-up of neurocysticercosis patients. Trans R Soc Trop Med Hyg 94: 673-676.

6. Harrison LJ, Joshua GW, Wright SH, Parkhouse RM (1989) Specific detection of circulating surface/secreted glycoproteins of viable cysticerci in *Taenia saginata* cysticercosis. Parasite Immunol 11: 351-370.

7. Guezala MC, Rodriguez S, Zamora H, Garcia HH, Gonzalez AE, et al. (2009) Development of a species-specific coproantigen ELISA for human *Taenia solium* taeniasis. Am J Trop Med Hyg 81: 433-437.

8. Nkouawa A, Sako Y, Li T, Chen X, Wandra T, et al. (2010) Evaluation of a loop-mediated isothermal amplification method using fecal specimens for differential detection of *Taenia species* from humans. J Clin Microbiol 48: 3350-3352.

9. Yamasaki H, Allan JC, Sato MO, Nakao M, Sako Y, et al. (2004) DNA differential diagnosis of taeniasis and cysticercosis by multiplex PCR.
J Clin Microbiol 42: 548-553.

10. Mayta H, Gilman RH, Prendergast E, Castillo JP, Tinoco YO, et al. (2008) Nested PCR for specific diagnosis of *Taenia solium* taeniasis. J Clin Microbiol 46: 286-289.

11. Willingham AL, 3rd, Engels D (2006) Control of *Taenia solium* cysticercosis/taeniosis. Adv Parasitol 61: 509-566.

12. Ito A, Plancarte A, Nakao M, Nakaya K, Ikejima T, et al. (1999) ELISA and immunoblot using purified glycoproteins for serodiagnosis of cysticercosis in pigs naturally infected with *Taenia solium*. J Helminthol 73: 363-365.

13. Sato MO, Yamasaki H, Sako Y, Nakao M, Nakaya K, et al. (2003) Evaluation of tongue inspection and serology for diagnosis of *Taenia solium* cysticercosis in swine: usefulness of ELISA using purified glycoproteins and recombinant antigen.
Vet Parasitol 111: 309-322.

14. Tsang VC, Pilcher JA, Zhou W, Boyer AE, Kamango-Sollo EI, et al. (1991) Efficacy of the immunoblot assay for cysticercosis in pigs and modulated expression of distinct IgM/IgG activities to *Taenia solium* antigens in experimental infections.
Vet Immunol Immunopathol 29: 69-78.

15. Sciutto E, Hernandez M, Garcia G, de Aluja AS, Villalobos AN, et al. (1998) Diagnosis of porcine cysticercosis: a comparative study of serological tests for detection of circulating antibody and viable parasites. Vet Parasitol 78: 185-194.
