# Supplementary material for: A Research Agenda for Helminth Diseases of Humans: Diagnostics for Control and Elimination Programmes
Source: PLoS Negl Trop Dis. 2012 Apr 24;6(4):e1601. doi: 10.1371/journal.pntd.0001601 (PMC3335877; doi:10.1371/journal.pntd.0001601)
Supplement: Text S6 — Diagnostics for Food-Borne Trematodiases. (DOCX) [file pntd.0001601.s006.docx]

**Text S6. Diagnosis of Food-Borne Trematodiases**

***Parasitological Diagnosis***

Parasitological diagnosis of clonorchiasis and opisthorchiasis (due to infection with *Clonorchis sinensis*, *Opisthorchis viverrini*, *O. felineus*, and other related fluke species) can be accomplished by demonstrations of eggs in faeces, bile or duodenal fluid. Although the Kato-Katz thick smear is the most commonly used technique, other methods such as Stoll's dilution or quantitative formalin ethyl acetate concentration can be used [[1-5](#_ENREF_1)]. Particular problems arise in parasitological diagnosis of zoonotic trematodes in South-East Asia due to the very similar appearances of trematode eggs, including those of *Clonorchis*, *Opisthorchis*, and other less common minute intestinal flukes [[6-8](#_ENREF_6)]. Definitive species diagnosis requires morphological identification of the adult flukes following expulsion, surgical resection, or at autopsy. Unfortunately, the issue of multiple species has largely been ignored in the literature, resulting in a likely overestimation of *C. sinensis* prevalence, and underestimation of the prevalence of other species, including minute intestinal flukes [[9](#_ENREF_9)]. As for other intestinal helminth infections, parasitological methods are insensitive in light infection. For example, in an autopsy study, faecal eggs were not detected in individuals harbouring less than 20 worms in the liver [[10](#_ENREF_10)].

***Antibody Tests***

Serodiagnostic tests have been developed for *Clonorchis* and *Opisthorchis* infections. As occurs in the filariases, these tests are relatively non-specific, and an antibody response can be detected long after curative treatment [[11-13](#_ENREF_11)]. Serological tests using recombinant antigens, and those detecting isotype-specific antibodies [[14](#_ENREF_14)] may be more specific. Reports of pilot assays using specific recombinant antigens for the diagnosis of *C. sinensis* or *O. viverrini* indicate that they are more sensitive and specific than tests using native parasite antigens [[15-17](#_ENREF_15)]. However, irrespective of antigen target, such tests do not enable differentiation between past and current infection.

***Antigen Detection***

Coproantigen diagnostic testing by ELISA with monoclonal antibodies raised against secretory antigens of intestinal and liver flukes have been developed [[18](#_ENREF_18),[19](#_ENREF_19)]. These showed a diagnostic sensitivity of 31–57% and a specificity of 70–100% [[18](#_ENREF_18),[19](#_ENREF_19)]. A monoclonal antibody-based copro-ELISA was found to be more sensitive than conventional faecal examination, particularly in light infections (< 500 eggs per gram) [[18](#_ENREF_18),[19](#_ENREF_19)]. The performance of copro-ELISAs in even lighter infections remains to be determined. Although data from copro-ELISA assays are promising a number of challenges remain before a simple diagnostic kit is developed, including maintaining a reliable supply of appropriate monoclonal antibodies.

***Molecular Diagnosis***

PCR-based tests for detection of fluke DNA in faeces appears to be an approach with some potential [[20-24](#_ENREF_20)]. Sensitivity of the detection at laboratory level has been reported as high as 2x10^-17^ ng DNA in faeces [[24](#_ENREF_24)]. However, results of tests undertaken with clinical stool samples have not always been in concordance with results of tests undertaken by standard egg detection [[25](#_ENREF_25)]. Several genes of food-borne trematodes such as mitochondrial DNA have been used for molecular diagnosis [[21](#_ENREF_21)]. Internal transcribed spacers (ITS1 and ITS2) have frequently been used as a molecular marker for differentiating closely-related food-borne trematode species because a high level of interspecies sequence divergence exists [[23](#_ENREF_23),[26](#_ENREF_26)]. ITS1-based tests are particularly useful for the discrimination of the human liver flukes and minute intestinal flukes, but less sensitive for diagnosis (~76%), whereas ITS2 is less specific but highly sensitive (~95%) for *O. viverrini* [[26](#_ENREF_26)]. Methods based on LAMP have been applied successfully for the detection of food-borne trematodes such as *C. sinensis* [[27](#_ENREF_27)], *Paragonimus westermani* [[28](#_ENREF_28)], *Fasciola hepatica,* *F. gigantica* [[29](#_ENREF_29)] and *O. viverrini* [[30](#_ENREF_30)]. The LAMP assay has the advantage of not requiring a thermocycler and allowing visual detection of DNA amplification by turbidity. Therefore, it represents an attractive diagnostic method for point-of-care testing. However, false positive tests can occur due to contamination. A potential drawback of all PCR-based diagnostic tests is the presence of PCR inhibitors that can reduce sensitivity of the test. Therefore, in general such tests are not currently ready for field deployment for clinical and epidemiologic purposes. Confirmation of which food-borne trematode species is the pathogen in question currently represents the most appropriate application of molecular techniques.

**References**

1. Bergquist R, Johansen MV, Utzinger J (2009) Diagnostic dilemmas in helminthology: what tools to use and when? Trends Parasitol 25: 151-156.

2. Ditrich O, Giboda M, Scholz T, Beer SA (1992) Comparative morphology of eggs of the Haplorchiinae (Trematoda: Heterophyidae) and some other medically important heterophyid and opisthorchiid flukes. Folia Parasitol (Praha) 39: 123-132.

3. Elkins DB, Haswell-Elkins MR, Mairiang E, Mairiang P, Sithithaworn P, et al. (1990) A high frequency of hepatobiliary disease and suspected cholangiocarcinoma associated with heavy *Opisthorchis viverrini* infection in a small community in north-east Thailand. Trans R Soc Trop Med Hyg 84: 715-719.

4. Hong ST, Choi MH, Kim CH, Chung BS, Ji Z (2003) The Kato-Katz method is reliable for diagnosis of *Clonorchis sinensis* infection. Diagn Microbiol Infect Dis 47: 345-347.

5. Viyanant V, Brockelman WY, Lee P, Ardsungnoen S, Upatham ES (1983) A comparison of a modified quick-Kato technique and the Stoll dilution method for field examination for *Opisthorchis viverrini* eggs. J Helminthol 57: 191-195.

6. Chai JY, Park JH, Han ET, Guk SM, Shin EH, et al. (2005) Mixed infections with Opisthorchis viverrini and intestinal flukes in residents of Vientiane Municipality and Saravane Province in Laos. J Helminthol 79: 283-289.

7. Kaewkes S, Elkins DB, Sithithaworn P, Haswell-Elkins MR (1991) Comparative studies on the morphology of the eggs of *Opisthorchis viverrini* and lecithodendriid trematodes. Southeast Asian J Trop Med Public Health 22: 623-630.

8. Radomyos P, Radomyos B, Tungtrongchitr A (1994) Multi-infection with helminths in adults from northeast Thailand as determined by post-treatment fecal examination of adult worms. Trop Med Parasitol 45: 133-135.

9. Utzinger J, Bergquist R, Olveda R, Zhou XN (2010) Important helminth infections in Southeast Asia diversity, potential for control and prospects for elimination.
Adv Parasitol 72: 1-30.

10. Sithithaworn P, Tesana S, Pipitgool V, Kaewkes S, Pairojkul C, et al. (1991) Relationship between faecal egg count and worm burden of *Opisthorchis viverrini* in human autopsy cases. Parasitology 102 Pt 2: 277-281.

11. Rim HJ (2005) Clonorchiasis: an update. J Helminthol 79: 269-281.

12. Ruangkunaporn Y, Akai PS, Chongsa-nguan M, Sri-Yeythong S, Kitikoon V, et al. (1994) Changes in serum antibodies to *Opisthorchis viverrini* in humans and hamsters following treatment of opisthorchiasis. Asian Pac J Allergy Immunol 12: 83-84.

13. Thammapalerd N, Tharavanij S, Nacapunchai D, Bunnag D, Radomyos P, et al. (1988) Detection of antibodies against *Opisthorchis viverrini* in patients before and after treatment with praziquantel. Southeast Asian J Trop Med Public Health 19: 101-108.

14. Wongratanacheewin S, Sermswan RW, Sirisinha S (2003) Immunology and molecular biology of *Opisthorchis viverrini* infection. Acta Trop 88: 195-207.

15. Hu F, Yu X, Ma C, Zhou H, Zhou Z, et al. (2007) *Clonorchis sinensis*: expression, characterization, immunolocalization and serological reactivity of one excretory/secretory antigen-LPAP homologue. Exp Parasitol 117: 157-164.

16. Ma C, Hu X, Hu F, Li Y, Chen X, et al. (2007) Molecular characterization and serodiagnosis analysis of a novel lysophospholipase from *Clonorchis sinensis*. Parasitol Res 101: 419-425.

17. Ruangsittichai J, Viyanant V, Vichasri-Grams S, Sobhon P, Tesana S, et al. (2006) *Opisthorchis viverrini*: identification of a glycine-tyrosine rich eggshell protein and its potential as a diagnostic tool for human opisthorchiasis. Int J Parasitol 36: 1329-1339.

18. Chaicumpa W, Ruangkunaporn Y, Kalambaheti T, Limavongpranee S, Kitikoon V, et al. (1991) Specific monoclonal antibodies to *Opisthorchis viverrini*.
Int J Parasitol 21: 969-974.

19. Sirisinha S, Chawengkirttikul R, Haswell-Elkins MR, Elkins DB, Kaewkes S, et al. (1995) Evaluation of a monoclonal antibody-based enzyme linked immunosorbent assay for the diagnosis of *Opisthorchis viverrini* infection in an endemic area.
Am J Trop Med Hyg 52: 521-524.

20. Duenngai K, Sithithaworn P, Rudrappa UK, Iddya K, Laha T, et al. (2008) Improvement of PCR for detection of *Opisthorchis viverrini* DNA in human stool samples. J Clin Microbiol 46: 366-368.

21. Le TH, Van De N, Blair D, Sithithaworn P, McManus DP (2006) Clonorchis sinensis and *Opisthorchis viverrini*: development of a mitochondrial-based multiplex PCR for their identification and discrimination. Exp Parasitol 112: 109-114.

22. Lovis L, Mak TK, Phongluxa K, Soukhathammavong P, Sayasone S, et al. (2009) PCR Diagnosis of *Opisthorchis viverrini* and *Haplorchis taichui* Infections in a Lao Community in an area of endemicity and comparison of diagnostic methods for parasitological field surveys. J Clin Microbiol 47: 1517-1523.

23. Traub RJ, Macaranas J, Mungthin M, Leelayoova S, Cribb T, et al. (2009) A new PCR-based approach indicates the range of *Clonorchis sinensis* now extends to Central Thailand. PLoS Negl Trop Dis 3: e367.

24. Wongratanacheewin S, Pumidonming W, Sermswan RW, Maleewong W (2001) Development of a PCR-based method for the detection of *Opisthorchis viverrini* in experimentally infected hamsters. Parasitology 122: 175-180.

25. Stensvold CR, Saijuntha W, Sithithaworn P, Wongratanacheewin S, Strandgaard H, et al. (2006) Evaluation of PCR based coprodiagnosis of human opisthorchiasis.
Acta Trop 97: 26-30.

26. Sato M, Thaenkham U, Dekumyoy P, Waikagul J (2009) Discrimination of *O. viverrini, C. sinensis, H. pumilio* and *H. taichui* using nuclear DNA-based PCR targeting ribosomal DNA ITS regions. Acta Trop 109: 81-83.

27. Cai XQ, Xu MJ, Wang YH, Qiu DY, Liu GX, et al. (2010) Sensitive and rapid detection of *Clonorchis sinensis* infection in fish by loop-mediated isothermal amplification (LAMP). Parasitol Res 106: 1379-1383.

28. Chen MX, Ai L, Zhang RL, Xia JJ, Wang K, et al. (2011) Sensitive and rapid detection of *Paragonimus westermani* infection in humans and animals by loop-mediated isothermal amplification (LAMP). Parasitol Res 108: 1193-1198.

29. Ai L, Li C, Elsheikha HM, Hong SJ, Chen JX, et al. (2010) Rapid identification and differentiation of *Fasciola hepatica* and *Fasciola gigantica* by a loop-mediated isothermal amplification (LAMP) assay. Vet Parasitol 174: 228-233.

30. Arimatsu Y, Kaewkes S, Laha T, Hong SJ, Sripa B (2012) Rapid detection of Opisthorchis viverrini copro-DNA using loop-mediated isothermal amplification (LAMP). Parasitol Int 61: 178-182.
